# Traceability and Anti-Counterfeiting in Agri-Food Supply Chains: A Review of RFID, IoT, Blockchain, and AI Technologies

**DOI:** 10.3390/s26051685

**Published:** 2026-03-06

**Authors:** Mohamed Riad Sebti, Ultan McCarthy, Anastasia Ktenioudaki, Mariateresa Russo, Massimo Merenda

**Affiliations:** 1 DIIES Department, University (Mediterranea) of Reggio Calabria, 89124 Reggio Calabria, Italy; riad.sebti@unirc.it; 2 Department of Land Sciences, South East Technological University, X91 Y074 Waterford, Ireland; ultan.mccarthy@setu.ie (U.M.); anastasia.ktenioudaki@setu.ie (A.K.); 3 Department of Agraria, University (Mediterranea) of Reggio Calabria, 89124 Reggio Calabria, Italy; mariateresa.russo@unirc.it; 4 HWA srl Spin-off Unirc Reggio Calabria, 89122 Reggio Calabria, Italy

**Keywords:** agri-food, traceability, anti-counterfeiting, internet of things, artificial intelligence, RFID, blockchain

## Abstract

By 2050, the global population is expected to reach approximately 10 billion, leading to a projected 50% increase in food demand relative to 2013 levels. If not adequately anticipated, this growing demand will place significant strain on agri-food systems worldwide, with disproportionate impacts on low- and middle-income countries. Moreover, current projections may underestimate the accelerating effects of climate change, political instability, and civil unrest, which continue to disrupt food production and distribution systems. In this context, technological advancements offer a promising pathway to enhance efficiency, improve transparency, and mitigate risks related to food safety, adulteration, and counterfeiting. Emerging innovations can decouple food production from environmental degradation while strengthening monitoring, verification, and accountability across supply chains. This review examines state-of-the-art technologies developed to support traceability and anti-counterfeiting in agri-food supply chains, considering their application across the full spectrum of stakeholders. To provide a system-level perspective, the review adopts a five-layer socio-technical traceability and anti-counterfeiting framework, comprising identity, sensing, intelligence, integrity, and interaction layers, which is used to map enabling technologies and reinterpret the evolution of traceability systems (TS 1.0–TS 4.0) as a progression of functional capabilities rather than isolated technological upgrades. Using this framework, the review analyzes the advantages and limitations of current solutions and clarifies how traceability and anti-counterfeiting functions emerge through technology integration. It further identifies gaps that hinder large-scale and equitable adoption. Finally, future research directions are outlined to address current technical, economic, and governance challenges and to guide the development of more resilient, trustworthy, and sustainable agri-food traceability systems.

## 1. Introduction

**Figure 1:**
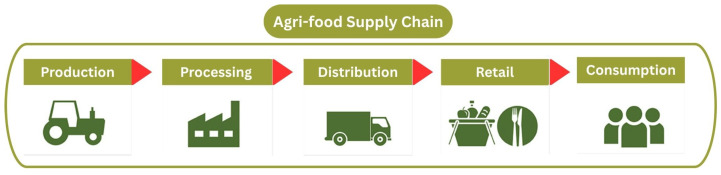
Agri-food supply chain.

In recent years, globalization patterns have dramatically altered our food habits, and as a result have also played a critical role in changing our global export and import channels and routes. These trends, coupled with the recent COVID pandemic, have emphasized the importance of our agri-food production systems and associated trading channels. Within these complex systems, services such as traceability and anti-counterfeiting (TRAC) have become crucial due to the valuable information they provide to customers and to stakeholders alike throughout the supply chain. Traceability is commonly defined as the ability to follow the movement of a food product one-step-forward and one-step-backward (OSF–OSB), as specified in Regulation (EC) No 178/2002 [1], creating a chain-wide series of linkages that establish the product history [2] and provide information about its origin and quality [3] across different stages of production, processing, and distribution. While this definition establishes essential regulatory compliance requirements, it does not fully capture the systemic role of traceability in modern, data-driven agri-food supply chains (AFSCs). In this review, traceability is therefore understood as the capability of a socio-technical system to continuously link physical products with verifiable digital records across all relevant stages of the supply chain, enabling the reconstruction, monitoring, and analysis of product history, handling conditions, and transformations. Under this perspective, traceability functions as a dynamic information infrastructure that supports visibility, accountability, and risk-aware decision-making across stakeholders. Traceability relies on three fundamental elements: (i) product identity (what the product is), (ii) event capture (what happens to it, where, and under which conditions), and (iii) information continuity (how these events are linked across time and organizational boundaries). Technologies such as barcodes, Radio-Frequency Identification (RFID), Near Field Communication (NFC), Internet of Things (IoT), Artificial Intelligence (AI), and blockchain do not independently constitute traceability; rather, they act as enabling components that support one or more of these elements. To structure this system-level perspective, this review adopts a layered conceptual framework for TRAC systems in AFSCs. The framework distinguishes five interdependent functional layers: an identity layer responsible for product identification and identity verification, a sensing layer that captures events and environmental conditions across the supply chain, an intelligence layer that interprets collected data through analytical and AI-based models, an integrity layer that ensures data trust, immutability, and auditability, and an interaction layer that enables verification, governance, and access to traceability information by stakeholders such as consumers, regulators, and certification bodies. These layers operate in a coordinated manner rather than as a linear pipeline, and system-level TRAC services emerge from their combined operation rather than from any single technology. Anti-counterfeiting refers to the illegal use of a registered trademark on similar products to deceive consumers into believing they are purchasing the original products [4]. When traceability systems are robust and consistently enforced across the supply chain, they create conditions that strongly support anti-counterfeiting by constraining unauthorized product substitution and enhancing detection capabilities. Nevertheless, authenticity can only be assured when traceability is complemented by reliable product identity verification methods. This review conceptualizes the boundary as follows: traceability provides continuous information continuity across supply chain events, while anti-counterfeiting specifically targets product authenticity through robust identity verification that prevents substitution or replication. Trust emerges when stakeholders believe this linkage between physical products and digital records is tamper-resistant (integrity layer) and independently verifiable (interaction layer), with authenticity representing confidence in the product’s true identity and origin. Historically, traceability was not systematically prioritized in agri-food systems, contributing to recurrent food safety incidents, product substitution, and limited visibility over transportation and storage conditions. Growing consumer demand for year-round and globally sourced food products has further intensified the need for reliable, real-time access to origin and handling information [5,6]. Limited traceability and information asymmetry have been shown to negatively affect consumer safety, economic stability, and regulatory compliance [7,8]. Moreover, the absence of effective traceability mechanisms has been linked to significant food waste, with estimates indicating that 30–50% of agri-food products may perish before reaching consumers [9,10]. These inefficiencies increase operational costs, erode consumer trust, and compromise product quality. Despite ongoing technological advancements, achieving reliable end-to-end TRAC across global AFSCs remains challenging due to complex logistics, preservation constraints, and regulatory heterogeneity. While technologies enabling farm-to-consumer tracking offer a clear pathway toward more resilient food systems, their practical implementation continues to face substantial technical, organizational, and economic barriers [11]. AFSCs are highly differentiated and complex and involve multi-variable stages [12], beginning with the farm, where raw agricultural products are cultivated. These products may or may not progress through a number of stages, including processing, packaging, distribution, and retail, before reaching the final consumer. Each stage of this supply chain requires monitoring and detailed record-keeping, which must be fully documented to ensure product integrity, safety, quality, and traceability. This process is shown in Figure 1, which illustrates the journey of agri-food products from the farm to the final consumer [13]. Numerous digital solutions, including IoT sensors, RFID/NFC, AI analytics, blockchain, and barcoding have been deployed to support end-to-end monitoring, tracing, and tracking across AFSCs. This review makes five unique contributions relative to prior work: (i) it adopts TRAC as the primary analytical focus, rather than reviewing technologies in isolation, thereby positioning technological solutions within an end-to-end TRAC system perspective, (ii) comprehensive multi-technology coverage analyzed through a unified five-layer TRAC framework, (iii) functional reinterpretation of the TS 1.0–4.0 evolution as a process of layered maturation, (iv) Technology Readiness Level (TRL)-based maturity assessment across all technologies, (v) smallholder-focused multi-criteria evaluation, together with an integrated analysis of ethics, privacy, and consumer participation in TRAC design. To further clarify the positioning and novelty of this review, Table 1 compares the present work with recent survey and review articles published in 2025 that address agri-food TRAC and related digital technologies. Unlike previous reviews that focus individually on blockchain-enabled traceability, IoT-based monitoring, or AI-driven transparency within AFSCs, this study provides an integrated, system-level interpretation of TRAC technologies. Existing surveys often evaluate technologies in isolation or emphasize a single architectural component, such as ledger security or sensor deployment, without systematically analyzing their interdependencies across supply chain stages and functional layers. In contrast, this review introduces a layered TRAC framework that links identity, sensing, intelligence, integrity, and interaction components into a unified analytical structure. By combining functional-layer mapping, TRL assessment, and a multi-criteria system evaluation (cost, scalability, reuse potential, traceability effectiveness, and counterfeit protection), this work moves beyond descriptive aggregation toward comparative synthesis. The review therefore contributes not only a comprehensive consolidation of the recent literature, but also a structured interpretive perspective that clarifies technological complementarities, deployment maturity, and system-level trade-offs within agri-food traceability ecosystems. Following the introduction, Section 2 describes the review methodology, including the literature search strategy, study selection process, analytical framework, and deployment maturity assessment. Section 3 discusses the historical evolution of traceability systems and compares regulatory practices across major regions. Section 4 examines the integration of AI and the IoT, first from a general perspective and then within agri-food applications. Section 5, Section 6, Section 7, Section 8 and Section 9 review the principal enabling technologies, where representative systems are analyzed and summarized. Section 10 provides a cross-technology comparison and system-level synthesis, with emphasis on cost, scalability, reuse potential, traceability effectiveness, and counterfeit protection for small and medium-sized actors. Section 11 outlines future research directions, and Section 12 concludes the paper.

## 2. Review Methodology

### 2.1. Review Design and Objectives

This study adopts a systematic scoping review methodology to map, categorize, and critically synthesize research on TRAC technologies in AFSCs. A scoping design was selected due to the multidisciplinary nature of the field, which spans sensing hardware, identification technologies, IoT infrastructures, AI, blockchain systems, and socio-technical governance aspects. The objective is not only to summarize existing solutions but also to analyze how heterogeneous technologies integrate across functional layers to enable end-to-end TRAC functionality and to assess their deployment maturity.

### 2.2. Information Sources

To ensure comprehensive coverage across engineering, computer science, and agri-food research domains, the literature was retrieved from multiple established scientific databases, including Scopus, IEEE Xplore, Web of Science, and Google Scholar. These sources were selected to capture both high-impact journal publications and conference proceedings relevant to sensing technologies, IoT systems, AI, blockchain, and traceability applications. Only peer-reviewed publications written in English were considered. The search covered the period 2005–2025 to reflect the evolution of traceability solutions from early barcode- and RFID-based systems to recent IoT, AI, and blockchain-enabled architectures. This time window defined the search scope only. Studies were included selectively based on their relevance to agri-food TRAC applications and their compliance with the eligibility criteria, rather than publication date alone. To further improve coverage, backward snowballing was conducted by screening the reference lists of eligible articles to identify additional relevant studies that may not have been captured through database queries. Although the search window spans 2005–2025, the majority of included studies were published after 2015. This temporal distribution reflects the natural technological evolution of TRAC systems. Earlier publications primarily focused on barcode- and RFID-based identification approaches, which provided basic product labeling and tracking capabilities but limited digital integration and analytics. In contrast, more recent years have seen the rapid emergence of IoT, blockchain, and AI-enabled architectures that support real-time sensing, secure data sharing, and intelligent decision-making, resulting in increased research activity. Importantly, study inclusion was guided by relevance to TRAC objectives rather than by specific technologies or publication periods. Consequently, both early barcode/RFID solutions and contemporary digital frameworks were considered, and the predominance of post-2015 studies reflects field maturation rather than selective filtering. The final literature search was conducted in May 2025.

### 2.3. Search Strategy

The search covered publications from 2005 to 2025, capturing the transition from early barcode-based systems to contemporary IoT, AI, and blockchain-enabled architectures. Representative search expressions combined TRAC concepts with agri-food and enabling-technology keywords:


(‘‘traceability’’ OR ‘‘provenance’’ OR ‘‘anti-counterfeiting’’ OR ‘‘authenticity’’)
AND (‘‘agri-food’’ OR agriculture OR ‘‘food supply chain’’)
AND (RFID OR NFC OR barcode OR QR OR sensor OR IoT
      OR blockchain OR AI OR ‘‘machine learning’’)

Equivalent query syntax was adapted for each database. Backward snowballing was additionally applied to references of relevant studies.

### 2.4. Eligibility Criteria

Studies were included if they satisfied all of the following conditions:

- Addressed traceability and/or anti-counterfeiting in AFSCs as a primary objective rather than as a secondary or peripheral application.
- Described the design, implementation, experimental evaluation, or deployment of a traceability- or anti-counterfeiting-related system, framework, or architecture.
- Incorporated at least one enabling technology within the TRAC stack (identity, sensing, intelligence, integrity, or interaction layers).
- Provided extractable technical information, such as system architecture, data acquisition mechanisms, communication protocols, analytical models, validation setup, or deployment environment.

Studies were excluded if they:

- Focused on non-agri-food sectors.
- Presented only high-level conceptual discussions, opinion articles, or policy analyses without system design, implementation, or validation components.
- Lacked sufficient architectural, methodological, or experimental detail to allow functional classification within the proposed TRAC framework.
- Were duplicates, grey literature, theses, reports, or non-peer-reviewed material.

To ensure consistency, studies were required to explicitly describe a concrete system architecture, implementation, or experimental validation. Papers mentioning traceability only conceptually without technical or operational detail were excluded. This criterion prevented inclusion of purely descriptive or policy-oriented works.

### 2.5. Study Selection Process

**Figure 2:**
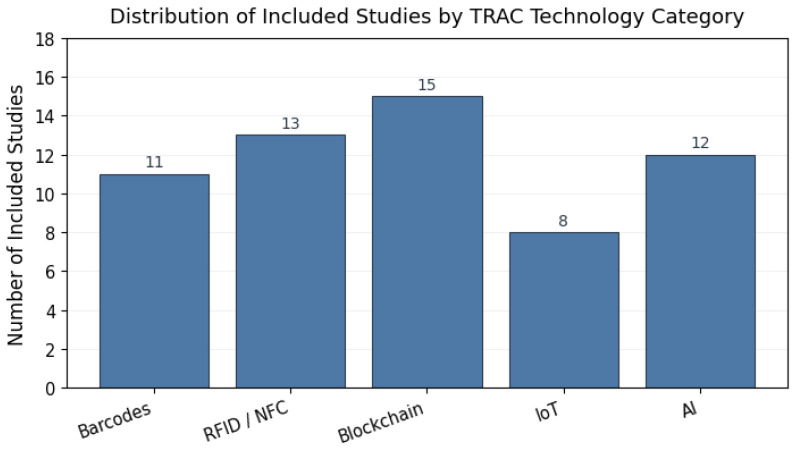
Distribution of included studies by primary TRAC technology category after the study selection process.

All records retrieved from the selected databases were exported and merged into a unified dataset. Duplicate entries were identified and removed prior to screening. The selection process was conducted in two stages. In the first stage, titles and abstracts were screened to eliminate clearly irrelevant studies based on domain mismatch, absence of traceability relevance, or lack of technical contribution. In the second stage, full-text articles were assessed against the predefined eligibility criteria to determine final inclusion. An initial set of approximately 250 records was identified across the selected databases. After deduplication, around 150 unique studies remained and were subjected to title and abstract screening. Studies that did not meet the eligibility criteria defined in Section 2.4, including lack of focus on agri-food traceability or anti-counterfeiting, absence of a technical system description, or insufficient methodological detail, were excluded at this stage. The remaining articles were then assessed through full-text review against the same eligibility criteria. This process resulted in a final corpus of 59 studies, which were included for qualitative synthesis, layer-based classification, and comparative analysis. The screening and selection process was conducted by the authors following a shared set of eligibility criteria (Section 2.4). To ensure consistency and reduce subjective bias, inclusion and exclusion decisions were cross-checked during the full-text review stage, and any ambiguities were resolved through discussion and consensus. This qualitative quality-control procedure was adopted to enhance the reliability and transparency of the review process. Figure 2 summarizes the distribution of the included studies across the main TRAC technology categories.

### 2.6. Data Extraction

For each included study, structured information was systematically extracted using a predefined data collection template. The extracted variables included:

- Agri-food domain or product category.
- Supply chain stage(s) addressed.
- Traceability and/or anti-counterfeiting functions.
- Enabling technologies employed.
- Sensing and data acquisition mechanisms.
- System architecture and data flow structure.
- Validation setting (simulation, laboratory experiment, pilot study, or real-world deployment).
- Reported technical, economic, organizational, or scalability limitations.

The extracted data supported functional classification within the proposed five-layer TRAC framework and enabled comparative maturity assessment.

### 2.7. Analytical Framework, Layer Classification, and TRL-Based Maturity Assessment

To enable structured cross-technology comparison and system-level interpretation, all included studies were analyzed using the five-layer TRAC framework. The framework decomposes traceability systems into the following functional layers:

1. Identity layer;
2. Sensing layer;
3. Intelligence layer;
4. Integrity layer;
5. Interaction layer.

For each study, the described system components were examined and mapped to one or more layers according to their primary functionality. Identification technologies were mapped to the identity layer, environmental sensors to the sensing layer, analytics and machine learning models to the intelligence layer, distributed ledgers or secure storage mechanisms to the integrity layer, and user-facing or governance mechanisms to the interaction layer. This functional classification enabled consistent comparison across heterogeneous architectures and facilitated identification of integration patterns, complementarities, and architectural gaps. In addition to functional mapping, the deployment maturity of each study was evaluated using the TRL scale. TRL was applied after study selection as an analytical metric rather than as an inclusion criterion, thereby allowing maturity assessment without biasing the screening process. The scale ranges from TRL 1 to TRL 9, where TRL 1–3 correspond to early-stage research and proof-of-concept development, TRL 4–6 represent laboratory validation and prototype demonstration in relevant environments, and TRL 7–9 indicate system demonstration and operational deployment under real-world AFSC conditions. To ensure consistency and reproducibility in TRL assignment, a rule-based rubric was adopted. TRL scores were assigned according to the highest level of validation explicitly reported in each study, following the criteria summarized in Table 2. The assessment was based exclusively on observable validation evidence such as simulation-based evaluation, laboratory prototypes, integrated system demonstrations, pilot deployments, or field implementations rather than on the underlying technology itself. As a consequence, similar technologies may be associated with different TRL values across studies, since TRL reflects deployment maturity rather than technological category. For example, RFID, AI, or blockchain-based solutions validated only through laboratory experiments were assigned lower TRLs than comparable technologies demonstrated with real products, stakeholders, or operational workflows. By combining layer-based functional classification with rule-based TRL assessment, the proposed methodology enables a two-dimensional synthesis of the literature, supporting both architectural comparison and evaluation of practical feasibility across traceability technologies.

### 2.8. Operationalization of Comparative Evaluation Criteria

To reduce the inherent subjectivity of qualitative multi-criteria comparisons, this review adopts explicit and context-driven operational definitions for each evaluation criterion used in the comparative analysis. Qualitative ratings (very low, low, medium, high, very high) were not assigned to technologies in isolation, nor based on abstract assumptions, but were derived from how each technology was implemented, evaluated, and discussed within the reviewed studies. For each criterion, ratings were accorded by comparing solutions employing the same technology across the literature and by examining the design choices, deployment contexts, and validation settings reported by the authors. The evaluation, therefore reflects observed usage and practical constraints rather than theoretical capabilities. Particular attention was given to whether solutions were explicitly designed for, or adapted to, AFSCs, which are characterized by heterogeneous actors, variable production scales, and cost-sensitive operations. Rather than introducing new quantitative measures, the qualitative assessment relies on implementation-related evidence commonly reported in the reviewed papers, such as system architecture, infrastructure requirements, deployment scale, reuse assumptions, and security mechanisms. These reported characteristics serve as practical proxies for comparison and enable consistent evaluation across heterogeneous technologies. The evaluation criteria were operationalized as follows:

- Cost: inferred from reported hardware or tag usage, infrastructure dependencies like cloud platforms, blockchain services, maintenance assumptions, and deployment scale described in the studies, particularly in relation to agri-food production contexts.
- Scalability: assessed based on whether the proposed solutions were demonstrated or discussed across multiple products, supply-chain stages, or operational contexts, and on the extent of infrastructure expansion implied by such scaling.
- Reuse potential: derived from whether system components were described as reusable across multiple products or traceability cycles, or instead treated as single-use or product-specific elements.
- Traceability effectiveness: evaluated based on the continuity and coverage of data capture reported across supply-chain stages, including how product identity, sensing data, and records were linked throughout the system.
- Counterfeit protection: inferred from the presence and role of authentication mechanisms, tamper-resistance features, and data integrity safeguards explicitly described in the system implementations.

These criteria were applied consistently across the reviewed studies based solely on information reported by the authors. While the resulting comparison remains qualitative, grounding the evaluation in documented implementation evidence enhances transparency, comparability, and analytical validity, supporting meaningful assessment of TRAC solutions in the agri-food sector.

## 3. Traceability over Years and Across Borders

**Figure 3:**
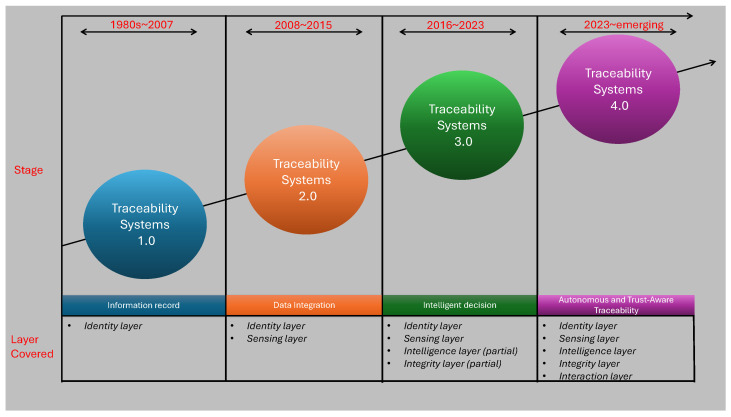
Evolution of traceability systems from TS 1.0 to TS 4.0 and associated functional layers.

Over the past two decades, Traceability Systems (TSs) have evolved significantly, driven by technological advancements and increasing demands for transparency and safety within AFSCs. As shown in Figure 3, this evolution can be interpreted not only as a progression of technologies, but also as a gradual enhancement of functional system capabilities across different traceability layers. Early traceability implementations, commonly referred to as TS 1.0 [22], were primarily based on fundamental record-keeping practices relying on paper-based or basic electronic documentation. From a functional perspective, TS 1.0 systems mainly addressed the identity layer by enabling basic product identification and OSF–OSB tracking, with limited or no support for real-time sensing, data interpretation, or integrity assurance. These systems fulfilled essential regulatory requirements and allowed rudimentary tracking and tracing of product movement, but remained constrained in terms of data richness, automation, and analytical capability. Despite their simplicity, TS 1.0 systems laid the structural foundation for subsequent developments. Around 2008, the emergence of the IoT marked the transition toward TS 2.0 [22], enabling electronic data integration across AFSCs. This generation expanded traceability functionality by strengthening the sensing layer through the deployment of connected sensors capable of capturing real-time environmental and logistical data. IoT technologies facilitated continuous monitoring of conditions such as temperature, humidity, and location, which became particularly critical for handling perishable products and improving food safety practices. While TS 2.0 significantly enhanced data availability and visibility, its analytical and integrity capabilities remained largely limited, with decision-making still dependent on human interpretation and centralized data-management architectures. Contemporary traceability systems have advanced toward TS 3.0 [22], characterized by the integration of AI and blockchain technologies to support more intelligent and trustworthy supply chain management. In TS 3.0, the intelligence layer is reinforced through AI-based data analytics, enabling predictive functions such as anomaly detection, quality assessment, and risk forecasting, while the integrity layer is strengthened through blockchain-based mechanisms that enhance data immutability, auditability, and accountability. This generation reflects a shift from reactive monitoring toward proactive and predictive traceability, addressing the growing demand for data-driven decision support and increased trust among stakeholders. From a system-level perspective, the evolution of traceability systems from TS 1.0 to TS 3.0 can be interpreted as a progressive maturation of layered functional capabilities rather than a simple succession of technologies. TS 1.0 primarily addressed the identity layer through basic product identification and OSF–OSB documentation. TS 2.0 extended this foundation by incorporating the sensing layer, enabling real-time data acquisition through IoT technologies. TS 3.0 further deepens system functionality by integrating intelligence layers, where AI supports predictive analytics and decision-making, together with integrity layers that leverage blockchain to ensure data immutability, trust, and auditability. This layered interpretation highlights that contemporary traceability systems differ not only in the technologies they employ, but in the depth of functional integration they achieve across the AFSC. More recently, traceability research has begun to identify the emergence of a new paradigm, commonly referred to as aTraceability System 4.0 (TS 4.0), which extends the principles of TS 3.0 by aligning traceability architectures with Industry 4.0 concepts [23,24]. While TS 3.0 systems leverage AI and blockchain to enhance intelligence and integrity at the system level, TS 4.0 places stronger emphasis on autonomy, decentralization, and context-aware decision-making across the supply chain. In this emerging generation, intelligence is increasingly embedded at the edge of the network through low-cost sensing devices and embedded AI, enabling on-device data processing, real-time risk assessment, and adaptive responses without continuous reliance on centralized cloud infrastructures. From a functional-layer perspective, TS 4.0 can be interpreted as a further maturation of the layered traceability model rather than a complete technological break. In addition to reinforcing the identity, sensing, intelligence, and integrity layers, TS 4.0 introduces advanced coordination and optimization capabilities that support predictive certification, proactive quality control, and dynamic compliance verification. Privacy-aware data-management strategies, including distributed learning and data minimization approaches, are increasingly explored to address scalability and confidentiality concerns in highly interconnected supply chains [23]. It is important to note that TS 4.0 should currently be regarded as an emerging and transitional phase, coexisting with advanced TS 3.0 deployments, rather than a fully standardized or universally adopted generation. Traceability regulations and system deployments vary significantly across regions, reflecting differences in regulatory priorities, market structures, and technological adoption. The systems implemented in China, the European Union (EU), and the United States (US) illustrate these regional distinctions and provide insight into the global evolution of traceability practices. In China, the establishment of the Food Safety Law (FSL) in 2015 has played a pivotal role in promoting secure AFSCs by enforcing rigorous traceability requirements [25]. The Chinese Food and Drug Administration (CFDA) oversees this regulatory framework across food production activities, while the National Health and Family Planning Commission (NHFPC) [26] conducts essential risk evaluations to uphold safety standards. These regulatory efforts primarily emphasize identity and sensing capabilities, ensuring that products can be identified and monitored throughout the supply chain. With approximately 52 specific rules currently in force [27], China has established a structured approach aimed at protecting public health and ensuring product quality across all stages of the food supply process [28]. Within the European Union, Regulation (EC) No 178/2002 [1] mandates that all food and feed operators document the origins and destinations of their products according to the OSF–OSB traceability model [29]. This framework requires systematic recording of supplier and customer information, along with delivery and transaction details, to support traceability across the supply chain. From a system perspective, the EU framework establishes a strong identity layer and is increasingly complemented by integrity-oriented mechanisms, such as the Rapid Alert System for Food and Feed (RASFF), which supports real-time information exchange among competent authorities. The RASFF infrastructure enhances responsiveness to food safety incidents and reinforces transparency and accountability across member states [30]. In the United States, traceability has gained increasing attention, particularly in the meat and livestock sectors, although a fully unified and mandatory national traceability framework has not yet been implemented. Instead, traceability initiatives continue to expand in response to consumer demand, export requirements, and international trade considerations [31,32]. US traceability systems emphasize supply chain efficiency and compliance with Country-of-Origin Labeling (COOL) requirements, focusing primarily on identity and documentation functions, while advanced sensing, intelligence, and integrity capabilities are adopted unevenly across sectors. As a result, traceability practices vary considerably among commodities, with consistent and standardized implementations for beef and poultry still under development [31,33]. Overall, regional approaches reflect different stages of functional maturity, shaped by regulatory objectives, technological readiness, and stakeholder expectations. Building upon the historical evolution of traceability systems illustrated in Figure 3, this review adopts a unified conceptual framework that decomposes agri-food TRAC architectures into five functional layers: identity, sensing, intelligence, integrity, and interaction. The progressive transition from TS 1.0 to TS 4.0 reveals that traceability systems evolved from simple information recording (identity layer) toward integrated architectures incorporating sensing, data analytics, trust enforcement, and stakeholder interaction mechanisms. Rather than evaluating barcodes, RFID, IoT, blockchain, and AI as isolated technologies, each solution is interpreted according to the functional role it performs within this layered structure. The identity layer provides product identification mechanisms such as barcodes, QR codes, RFID, and DNA markers. The sensing layer captures environmental and process-related events through IoT sensors and monitoring devices. The intelligence layer applies analytics and AI models to transform raw data into actionable insights. The integrity layer ensures data trustworthiness and immutability through blockchain or cryptographic mechanisms. Finally, the interaction layer enables stakeholder access, verification, and governance through mobile applications, dashboards, and consumer-facing interfaces. This layered abstraction is not proposed as a prescriptive implementation standard, but as an analytical tool derived from recurrent functional patterns observed across the reviewed literature. All surveyed studies can therefore be classified according to the layer(s) they support, enabling systematic cross-technology comparison, identification of complementarities, and detection of architectural gaps. By mapping technologies to functional responsibilities rather than device categories, the framework provides a system-level perspective that extends beyond technology-centric descriptions.

## 4. Artificial Intelligence and Internet of Things in the Agri-Food Sector

**Figure 4:**
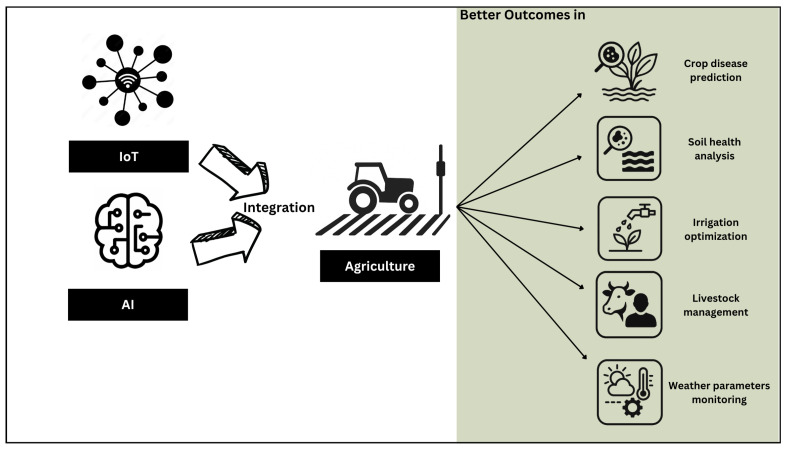
The results of the integration of AI and IoT in agriculture.

Within the five-layer TRAC framework, IoT and AI serve distinct but complementary roles. IoT constitutes the sensing layer infrastructure, providing distributed data acquisition (what/where/when) through sensors, RFID, and connectivity. AI operates at the intelligence layer, performing analytical interpretation (why/how/what-next) through pattern recognition, prediction, and decision support. Their TS 3.0 synergy emerges when IoT feeds real-time data streams to AI models, enabling proactive traceability rather than reactive monitoring, while neither replaces the identity, integrity, or interaction layers. The agri-food sector and all its outputs are an essential component of modern-day society and, as a result, demand considerable time and resources to build an efficient supply chain. A modern day fit-for-purpose supply chain must balance affordability, sustainability, and adequacy to uphold traceability and reduce counterfeiting. Deploying, modernizing and enhancing the AFSC calls for innovative approaches, as advancements in traceability depend on these improvements. Recent strides in integrating systems have greatly supported an array of industries, highlighting the need for seamless communication among system components and across geographic territories. IoT is a key approach that facilitates communication among devices, sensors, and other entities. IoT has revolutionized a number of sectors, including environmental monitoring [34,35], infrastructure management [36], industrial applications, and healthcare [37]. It has also been successfully deployed across a number of additional applications, including smart homes [38], smart cities [39], smart energy [40], autonomous vehicles [41], campus management [42], logistics [43], and smart agriculture, where its key applications include UAV farming, monitoring farm conditions, precision farming, supply chains management, tracking and tracing [44], monitoring forestry, aquaponics farms, and analytic data prediction [45,46,47]. Many of these advancements are largely due to the integration of AI [48,49]. Over the past few decades, AI has emerged as a prominent research topic [50,51], rapidly evolving and being applied across numerous industries for decision-making, automation, classification, etc. In TRAC systems, AI is primarily used to analyze sensing data and support predictive and decision-making functions. AI identifies patterns that facilitate automation and provide insights. For instance, in healthcare, AI can analyze medical records to assist in diagnosing diseases [52], enhancing efficiency and accuracy in medical settings. In terms of reasoning, AI systems are designed to analyze situations and solve problems in real-time. In finance, AI detects fraudulent activities by recognizing unusual transaction patterns [53]. This real-time analysis helps organizations mitigate risks and safeguard assets. When it comes to decision-making, AI leverages data to make informed choices in real or near real-time. This capability is demonstrated in applications such as autonomous driving [54], where vehicles must respond quickly and safely to dynamic road conditions. The integration of these capabilities not only supports human efforts but also significantly enhances outcomes across various sectors, from healthcare to transportation and finance. The integration of IoT with AI has the potential to revolutionize multiple industries [55], creating a powerful technology that addresses various challenges across different fields. By processing and analyzing the vast amounts of data collected by IoT sensors, AI can generate actionable insights. Moreover, AI enables IoT devices to interact with external sources effectively. In the agri-food sector, the integration of AI with IoT has proven to be highly advantageous [56]. It provides farmers with critical insights into crop health, soil conditions [57], livestock management, and weather parameters such as humidity and temperature [58]. This integration also improves irrigation practices and water quality management by enabling real-time monitoring of environmental parameters such as soil moisture, temperature, and humidity, which are critical for efficient water use. AI algorithms facilitate the efficient handling of data collected from IoT devices, sensors, and drones, allowing for rapid and accurate data analysis. Based on this analysis, AI can optimize irrigation schedules [59,60], predict crop diseases [61], and provide precise recommendations for fertilizer and pesticide applications [62]. These capabilities lead to reduced resource waste, more targeted interventions, and ultimately increased agricultural yields. The integration of IoT and AI aims to enhance various aspects of the agri-food sector, including ensuring food quality and safety, minimizing environmental impact, and providing monitoring information throughout the growth, harvesting, and transportation processes. The promise of AI and IoT integration lies in its potential to transform supply chain management, offering solutions to address food security, sustainability, efficiency, traceability, and anti-counterfeiting challenges. These benefits can be viewed collectively as better outcomes resulting from the combined use of AI and IoT, as illustrated in Figure 4.

## 5. Barcode, Non-Electronics Approaches and Molecular-Based Traceability

**Figure 5:**
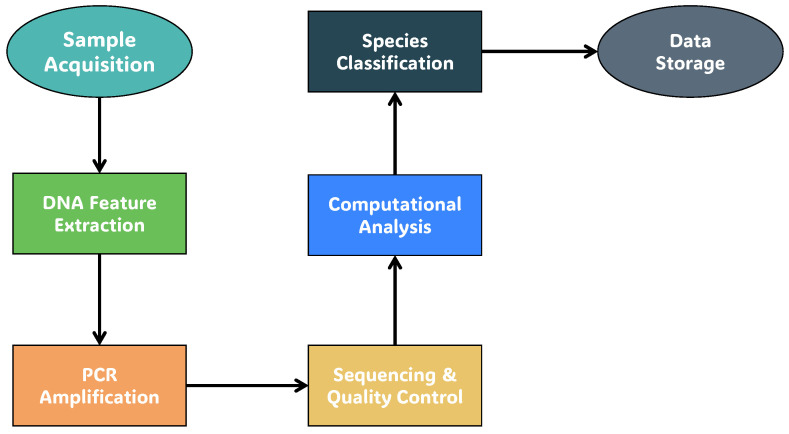
Workflow of DNA barcoding.

**Figure 6:**
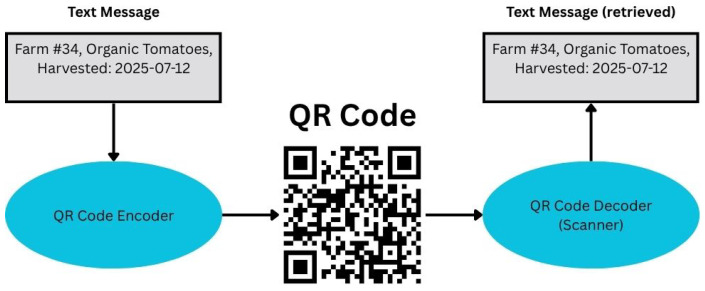
QR code encoding and decoding.

Traditional food traceability approaches have relied on physical barcodes, labels, and other non-electronic identifiers. However, these methods have limitations in terms of durability, security, and the ability to provide detailed information about a product’s origin and authenticity. In recent years, various techniques have emerged as powerful tools for food traceability and authentication, including barcoding, DNA barcoding [63], with the flow diagram for DNA barcoding [64], shown in Figure 5, and QR codes [65]. The use of QR codes, as shown in Figure 6, involves encoding information into a scannable image, which can then be decoded to retrieve the original information [66], making it a robust method for product identification and traceability. From a system-level perspective, the technologies reviewed in this section primarily contribute to the identity and interaction layers of agri-food TSs. Barcodes and QR codes enable product-level identification and provide user-facing access points for traceability information, facilitating verification by consumers and other stakeholders. DNA barcoding extends identity verification by introducing biological authentication, allowing the detection of species-level fraud that cannot be identified through visual or chemical inspection alone. However, these technologies do not inherently capture real-time events, environmental conditions, or data integrity guarantees, and therefore require integration with sensing, intelligence, and integrity layers to support comprehensive, end-to-end traceability. Several QR-based traceability platforms have been proposed across different agri-food contexts. These systems generally couple product-level identifiers with cloud or web backends to digitize production records and provide consumer-facing transparency. Representative examples include blockchain-integrated QR solutions [67,68], low-cost batch tracking platforms for dairy enterprises [69], web-service architectures for vegetable supply chains [70], large-scale anti-counterfeiting deployments [71], and cloud-based TSs for olive oil and regional consortia [72,73]. While these implementations demonstrate feasibility and affordability, most remain constrained by cloud dependency, limited sensing integration, scalability concerns, or prototype-level validation. Within the SISTABENE project, a multi-product traceability platform integrating product identification and data exchange among farmers was proposed [74]. Although conceptually robust, the framework remained a prototype without industrial-scale validation. DNA barcoding has been widely investigated as a molecular authentication technique for detecting species substitution and product fraud across diverse agri-food categories. Applications include species identification in commercial tea products, floral-origin tracing in honey, mislabeling detection across seafood and botanical products, and adulteration analysis in fruit juices [63,75,76,77,78]. While DNA-based approaches provide strong biological verification that surpasses visual or chemical inspection, they typically require laboratory infrastructure, controlled sample preparation, and specialized expertise, which may limit scalability and real-time deployment within operational supply chains. QR codes and DNA barcoding have emerged as complementary tools for enhancing transparency and authenticity at the product identity level, particularly when integrated with backend systems that support data capture, analytics, and integrity assurance. QR codes offer cost-effective, consumer-friendly access to product data, especially when integrated with blockchain or authentication systems [67,68], while DNA barcoding provides biological verification that enables reliable species identification and fraud detection across diverse food products [75,76,77,78]. Despite their effectiveness, most implementations remain prototype-level, facing challenges related to scalability, data accuracy, and operational costs. Both technologies also introduce important ethical and privacy considerations. QR-based systems may collect scan metadata or enable linkability between users and products, requiring the use of opaque identifiers, encrypted connections, and clear data-retention policies [79]. DNA barcoding, on the other hand, involves handling biological material, which necessitates informed consent, a documented chain of custody, and transparent reporting of analytical uncertainty. Establishing responsible data governance and bioethical safeguards will be essential to ensure that these technologies deliver trustworthy, secure, and socially acceptable traceability across AFSCs. The reviewed works are summarized in Table 3.

Barcode- and QR-based approaches provide low-cost, line-of-sight identification using printed labels that can be decoded through commodity scanners or smartphones, making them highly accessible for small and medium-sized producers. DNA barcoding complements these techniques by enabling molecular-level authentication through laboratory analysis. Compared with RFID or IoT sensing infrastructures, these technologies require minimal hardware investment and energy consumption but lack automated, real-time data capture capabilities. From an operational perspective, their primary advantages include affordability, simplicity, and high deployment maturity, which support rapid adoption across diverse AFSC contexts. However, they remain vulnerable to manual handling errors, label tampering, and limited data granularity. DNA-based methods strengthen authenticity verification but introduce higher costs, specialized equipment requirements, and slower processing times, reducing suitability for continuous or large-scale monitoring. Within the proposed TRAC framework, these technologies primarily support the identity and interaction layers by enabling product labeling and consumer-facing verification, while DNA barcoding enhances biological identity assurance. Barcode- and DNA-based approaches should be regarded as foundational identification mechanisms that require integration with sensing, intelligence, and integrity technologies to achieve comprehensive end-to-end traceability.

## 6. RFID and NFC Approaches

**Figure 7:**
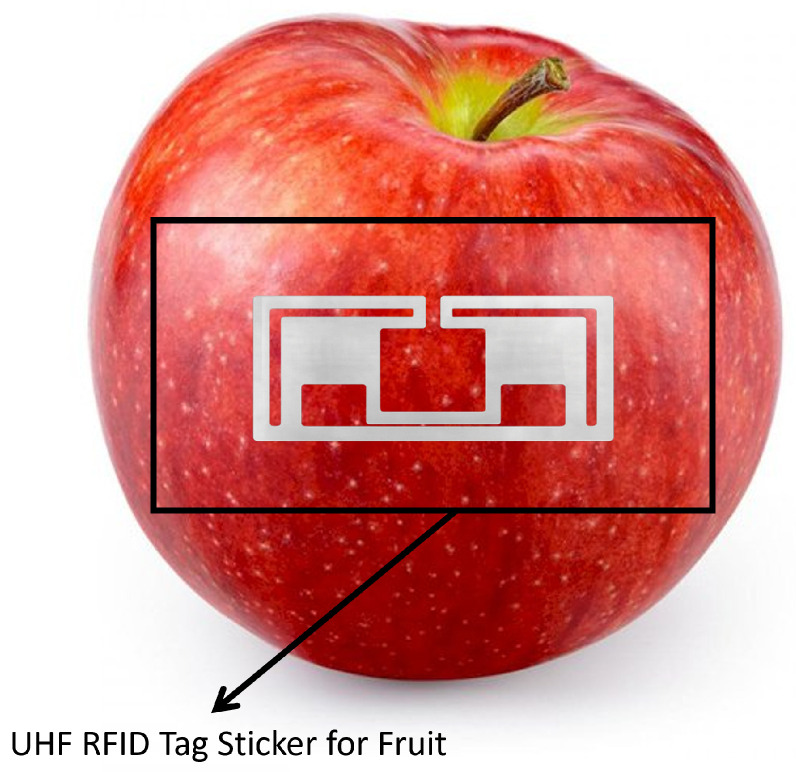
Example of UHF RFID tag applied to an apple for automated tracking and billing.

RFID and NFC are technologies used for wireless data transmission and object tracking across many domains [80,81], including logistics, healthcare, retail, and more. RFID uses electromagnetic fields to automatically identify and track tags over short to long distances, while NFC, a subset of RFID, operates at much shorter ranges and is commonly used for tasks like contactless payments and product authentication. In the agri-food sector, both RFID [82] and NFC [83] have been applied to enhance various processes, such as improving traceability [84], ensuring product authenticity, and increasing transparency throughout the supply chain, from farm to consumer. To avoid a purely technology-centric interpretation, RFID and NFC are examined here from a system-level traceability perspective. Within the layered TRAC framework adopted in this review, RFID and NFC primarily support the identity layer by enabling unique product identification and automated tracking, while extensions with environmental sensors contribute to the sensing layer. In consumer-facing scenarios, NFC further supports the interaction layer by enabling direct access to traceability information through personal devices. Their effectiveness, therefore, depends not on the communication technology alone, but on how they are integrated with data management, analytics, and integrity mechanisms across the overall traceability system. Figure 7 shows an example of an RFID tag attached to an apple [85], highlighting the use of UHF RFID technology for fruit labeling. The tag maintains impedance matching on high-water-content surfaces like apples, ensuring reliable tracking and automated billing in retail environments, with a read range of up to 3 m. While tagging each fruit with an RFID enhances traceability, the cost per unit makes it impractical for large-scale, low-value produce. From a traceability architecture standpoint, these limitations highlight that RFID- and NFC-based solutions are rarely sufficient as standalone systems and require complementary intelligence- and integrity-layer components to achieve end-to-end transparency and trust. Numerous RFID- and NFC-based traceability architectures have been explored across storage, logistics, and consumer-facing stages of the AFSC. Representative implementations include blockchain-integrated RFID frameworks for end-to-end transparency [6,86], warehouse and bin-level tracking systems for internal logistics [87,88], environmental monitoring solutions combining RFID with temperature, gas, or freshness sensors [89,90,91], smartphone-enabled NFC platforms for consumer interaction [92,93], and security-enhanced architectures employing cryptographic mechanisms [94,95]. While these approaches demonstrate the versatility of RFID/NFC technologies across multiple operational contexts, most remain constrained by infrastructure cost, sensing accuracy limitations, energy consumption, or prototype-level validation, which restrict large-scale adoption. From a sensing and hardware perspective, practical RFID/NFC deployments must also address physical-layer reliability constraints. Tag read accuracy, antenna orientation, signal attenuation near high-moisture or metallic products, collision handling in dense tag environments, and reader coverage density directly affect data completeness and temporal resolution. In addition, battery-assisted or sensor-integrated tags introduce energy and maintenance constraints that influence long-term stability. These engineering factors can lead to missed reads, duplicated events, or inconsistent timestamps, which propagate to higher-layer analytics and integrity mechanisms if not properly mitigated. Therefore, careful network planning, redundancy, calibration, and fault-tolerant acquisition strategies are essential for dependable large-scale operation in real agri-food environments. Moreover, the wireless and data-sharing nature of RFID and NFC introduces ethical and privacy concerns [96,97,98]. Unauthorized access to tag data could expose sensitive information about producers and supply routes [99,100], while continuous tracking may raise fears of surveillance and data misuse. To safeguard stakeholders, robust data governance, encryption standards, and transparent consent policies are essential [96,98,99]. Implementing these measures will ensure that RFID and NFC systems remain secure, privacy-respecting, and socially acceptable tools for advancing TRAC in AFSCs. Overall, the reviewed studies in Table 4 demonstrate that RFID and NFC technologies are most effective when embedded within multi-layer traceability architectures rather than deployed in isolation. RFID excels in automated identification and internal logistics monitoring, whereas NFC is particularly suited for consumer interaction and point-of-sale verification. However, neither technology alone guarantees data integrity or trust; robust TRAC systems emerge only when identity and sensing functions are coupled with integrity and intelligence mechanisms and higher-level intelligence for validation and decision support.

RFID and NFC technologies enable automated, non-line-of-sight identification and wireless data exchange, providing faster and more reliable tracking than barcode-based approaches. RFID supports bulk reading, longer communication ranges, and integration with environmental sensors, making it suitable for warehouse automation and logistics monitoring, whereas NFC favors short-range, low-power interactions that are well suited for consumer authentication and point-of-sale verification. Compared with purely optical identifiers, these technologies improve automation and reduce manual intervention but introduce higher hardware costs, energy demands, and infrastructure complexity. From an operational perspective, RFID/NFC systems offer real-time tracking, sensor integration, and improved process efficiency; however, tag cost, reader deployment, interoperability challenges, and maintenance requirements often limit feasibility for low-margin agri-food products. Furthermore, neither RFID nor NFC inherently guarantees data integrity or trust, requiring integration with intelligence and integrity-layer technologies such as analytics platforms or blockchain ledgers. Within the proposed TRAC framework, RFID and NFC primarily contribute to the identity and sensing layers, while NFC additionally strengthens the interaction layer through direct consumer access. RFID/NFC should be considered enabling infrastructure components that complement, rather than replace, higher-layer mechanisms for analytics, validation, and secure data management.

## 7. Blockchain and Ledger Technology Approaches

**Figure 8:**
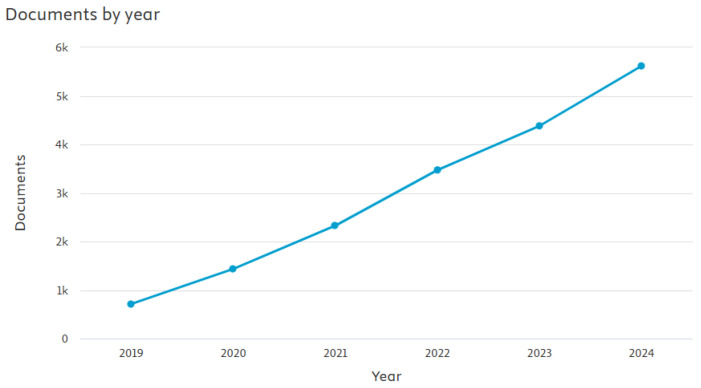
Annual growth of blockchain-related publications (2019–2024).

Blockchain, as defined in Section 5, has consistently proven to be an effective approach due to its decentralized and tamper-evident architecture. In recent years, the number of blockchain-based publications has grown linearly [101] as shown in Figure 8. It is now considered one of the most widely adopted technologies for real-time data logging, offering secure, transparent, and immutable records. From a layered traceability perspective, blockchain primarily reinforces the integrity layer by ensuring data immutability, auditability, and shared trust among stakeholders, while indirectly supporting the identity layer through secure linkage of product identifiers to verifiable digital records. These features make blockchain particularly valuable in TSs across many sectors, including agri-food, pharmaceuticals, and logistics, where maintaining an auditable and trustworthy chain of information is essential. Traceability data can be stored and shared securely through a collaborative blockchain network [102] involving farmers, processors, distributors, and retailers. This collaborative blockchain can be viewed as global, much like cryptocurrency blockchains [103]. As blockchain technology becomes increasingly adopted across various sectors [104], many researchers have focused on the applications of ledger and blockchain technologies. Numerous blockchain-enabled traceability architectures have been proposed across cultivation, processing, logistics, and retail stages of the AFSC. A dominant design pattern integrates blockchain with IoT sensing infrastructures to enable automated, tamper-resistant capture of environmental and operational data [105,106,107,108,109]. In these systems, IoT and RFID components primarily operate at the sensing and identity layers, while blockchain functions as the integrity layer by ensuring immutability, auditability, and cross-organizational trust. To address blockchain storage and throughput limitations, several studies adopt hybrid on-chain/off-chain architectures, frequently leveraging Interplanetary File System (IPFS) [110] or distributed file systems to store large sensor datasets, images, or transaction records while maintaining cryptographic hashes on-chain [111,112,113,114]. This approach improves scalability and storage efficiency but introduces synchronization, governance, and latency challenges under real-time conditions. Consumer-facing verification is commonly supported through QR code or RFID integration, allowing stakeholders to access blockchain-backed traceability records via mobile applications or web interfaces [115,116,117,118,119]. Permissioned blockchain frameworks, such as Hyperledger Fabric, are frequently employed to enhance access control, privacy preservation, and regulatory compliance [114,117,120], albeit at the cost of increased infrastructure complexity and centralized governance requirements. While some architectures incorporate additional intelligence-layer components, such as data filtering or AI-based preprocessing prior to blockchain storage [107], the majority remain focused on secure data recording rather than advanced analytics or decision support. Overall, blockchain-based TSs consistently demonstrate improved transparency, tamper resistance, and accountability compared with centralized databases. However, most reviewed implementations remain limited to prototypes, pilot deployments, or simulated environments, with persistent challenges related to infrastructure cost, energy consumption, interoperability, latency, and system governance. These limitations are particularly pronounced for small and medium producers, reinforcing the observation that blockchain is most effective when deployed as an integrity-layer component within a broader, multi-layer TRAC architecture rather than as a standalone traceability solution. Recent advances in blockchain-based research highlight its role as a key enabler of transparency and trust in agri-food traceability. Within the proposed layered framework, blockchain does not operate as a standalone traceability solution, but rather as an enabling integrity layer that must be combined with identity, sensing, and intelligence layers to achieve end-to-end TRAC functionality in AFSCs. Most systems achieve secure and verifiable data exchange, particularly when integrated with IoT, RFID, and IPFS technologies. However, many implementations remain at conceptual or pilot stages, often validated only through simulations or limited-scale trials. Persistent challenges include scalability, interoperability, energy efficiency, and deployment cost, with limited evidence of sustained field performance across diverse supply chains. Ethical and privacy dimensions are equally critical in blockchain-enabled traceability. While blockchain’s immutability and transparency enhance accountability throughout the agri-food network [101,121,122], they may also expose sensitive operational data, raising privacy concerns for farmers, distributors, and retailers [101,123,124]. Permissioned blockchains [125] and off-chain storage solutions are often adopted to mitigate these risks, yet further research is needed to strike a balance between transparency and confidentiality, especially for small-scale producers aiming to participate in decentralized ecosystems. In summary, blockchain-driven solutions substantially enhance traceability, transparency, and anti-counterfeiting within agri-food systems. The reviewed works are summarized in Table 5.

Blockchain technologies provide a distributed, tamper-evident ledger that strengthens the integrity layer of the proposed TRAC framework by ensuring immutability, shared trust, and auditability of recorded events. Unlike identification or sensing technologies, blockchain does not directly capture physical data; rather, it secures and validates information generated by identity and sensing layers such as RFID, QR, and IoT sensors. Consequently, its effectiveness depends on reliable upstream data acquisition and appropriate system integration. From an operational standpoint, blockchain enhances transparency and accountability across multiple stakeholders, supporting provenance verification, anti-counterfeiting, and automated compliance through smart contracts. However, practical deployment remains constrained by transaction latency, storage overhead, energy consumption, governance complexity, and infrastructure cost, particularly for small and medium-sized producers. Blockchain should be regarded as an enabling integrity mechanism that complements, rather than replaces, identity, sensing, and intelligence technologies within end-to-end agri-food traceability architectures.

## 8. IoT Sensor Approaches

**Figure 9:**
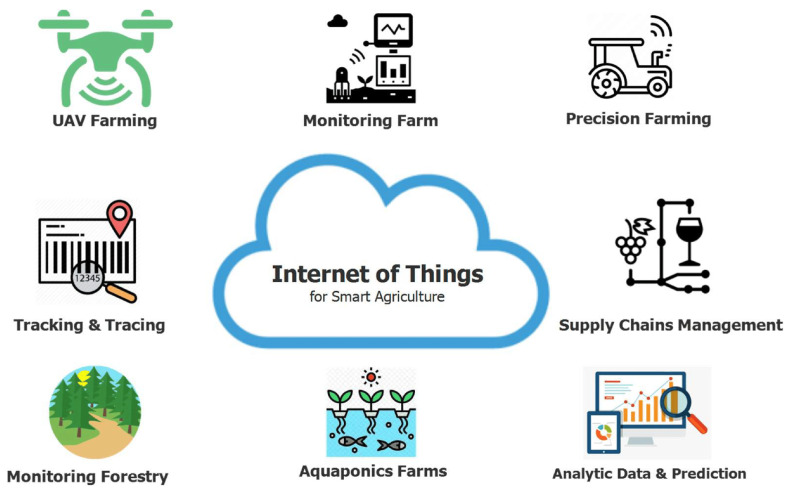
The uses of IoT in agriculture [126].

Figure 9 illustrates the diverse applications of IoT technologies in the agricultural domain, highlighting their growing role across various smart farming practices [126]. The uptake and adoption of IoT devices and technologies across agri-food applications has recently increased exponentially. This has affected the AFSCs products, sparking significant interest in research and innovation for the creation of trustworthy, auditable, and transparent TSs. Within the layered TRAC framework adopted in this review, IoT technologies primarily operate at the sensing layer, enabling continuous capture of environmental, logistical, and process-related events across AFSCs, while also supporting downstream intelligence and integrity functions through data integration. Due to the fact that current IoT-based traceability and provenance systems for AFSCs are constructed on top of centralized infrastructures, there are still many problems that need to be resolved as well as significant risks, such as single points of failure and data integrity issues [127,128]. Numerous IoT-based traceability architectures have been explored to enable continuous sensing and real-time monitoring across cultivation, storage, transportation, and retail stages of AFSCs. Representative designs integrate heterogeneous sensor networks with middleware or cloud platforms for data aggregation [129], combine IoT with blockchain or distributed ledgers to secure captured events [130,131,132], employ low-power communication technologies such as LoRaWAN or fog computing for scalable field deployments [133], and utilize greenhouse or controlled-environment sensing for quality assurance and consumer transparency [134,135]. Additional solutions incorporate RFID identification and cloud dashboards for product-level monitoring [136]. While these approaches demonstrate the feasibility of real-time, end-to-end visibility, most remain limited to pilot or small-scale environments and often face challenges related to connectivity reliability, energy consumption, cybersecurity, and infrastructure cost. When IoT is combined with blockchain, RFID, or QR codes, IoT significantly strengthens data transparency, anti-counterfeiting, and operational efficiency [130,131,133,134]. Despite these advances, most systems remain pilot-scale or laboratory-tested, with limited validation in large-scale, heterogeneous, or cross-border networks. Common barriers include connectivity reliability, energy constraints, and integration costs, as well as insufficient security and governance mechanisms to safeguard sensitive data. At the same time, large-scale IoT deployments raise ethical and privacy challenges, as they may expose confidential farm or operational information and increase vulnerability to cyberattacks [137,138,139]. Insecure or poorly managed IoT devices can compromise both data integrity and user trust. Mitigating these risks requires secure communication protocols, data anonymization, and transparent governance frameworks to ensure accountability and regulatory compliance. From a sensing-engineering perspective, the effectiveness of IoT-based TRAC systems depends critically on the reliability of the underlying sensor infrastructure. Measurement accuracy, calibration stability, sampling frequency, synchronization across distributed nodes, packet loss, and communication latency directly influence data completeness and temporal resolution. In many reviewed implementations, low-cost sensors and wireless links introduce noise, missing values, or drift, which can propagate to intelligence and integrity layers if not properly filtered or validated. Therefore, robust edge processing, redundancy mechanisms, fault detection, and secure data acquisition protocols are essential to ensure that sensing-layer data remain trustworthy before being stored, analyzed, or exposed to stakeholders. Overall, IoT-driven TSs substantially enhance transparency, authenticity, and trust within AFSCs, but their success depends on addressing the persistent technical, ethical, and organizational challenges that limit widespread, secure adoption. However, without robust integration with the intelligence and integrity layers, IoT-based TSs remain vulnerable to data overload, security breaches, and limited interpretability, reducing their effectiveness in large-scale and multi-actor AFSCs. The reviewed works are summarized in Table 6.

IoT technologies constitute the primary sensing infrastructure of the proposed TRAC framework by enabling automated capture of environmental, logistical, and process-related events. Compared with barcode or RFID solutions, IoT provides richer, continuous, and multi-parameter data streams that support condition monitoring, predictive analytics, and quality assurance. However, these benefits come at the cost of higher system complexity, energy requirements, and dependence on reliable communication networks. From an operational perspective, IoT deployments improve visibility and decision-making but introduce challenges related to device management, interoperability, cybersecurity, and large-scale data handling. Moreover, without complementary integrity mechanisms, sensed data remain vulnerable to tampering or loss, underscoring the need for integration with blockchain or secure back-end platforms. IoT should be viewed as an enabling sensing layer that must be tightly coupled with identity, intelligence, and integrity technologies to achieve robust, trustworthy, and scalable end-to-end traceability.

## 9. AI Approaches

**Figure 10:**
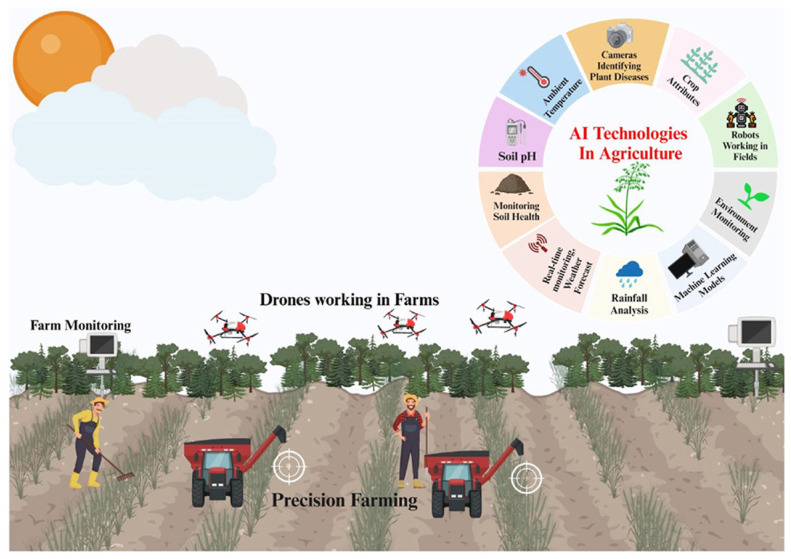
AI technologies in agriculture [164].

**Figure 11:**
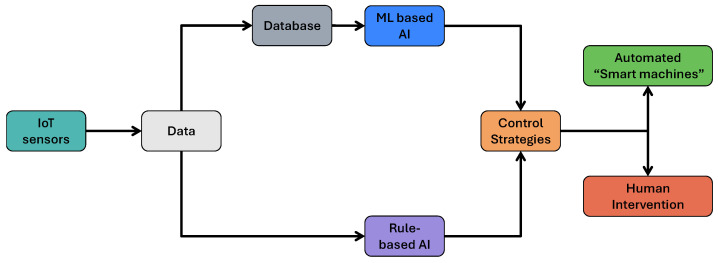
An example of a system architecture based on the integration of IoT with AI.

As discussed in Section 4, AI and IoT play a critical role in modernizing the agri-food sector. Building on their general benefits, recent efforts have increasingly focused on using AI to strengthen traceability and combat counterfeiting across the AFSCs. By analyzing real-time data from sensors, drones, and other IoT devices as shown in Figure 10. AI enables reliable product tracking, anomaly detection, and source verification [140,141,142,143]. In many cases, system architectures [140] follow a model like the one shown in Figure 11. Within the layered TRAC framework adopted in this review, AI primarily operates at the intelligence layer, where it transforms raw traceability data into actionable knowledge through prediction, classification, anomaly detection, and decision-support functions. Numerous AI-driven traceability architectures have been proposed to enhance authenticity verification, quality prediction, anomaly detection, and supply–demand optimization across AFSCs. Representative approaches include Deep Learning (DL) models for spectral or image-based product authentication [144,145], gradient-boosting and Recurrent Neural Networks (RNN) for freshness prediction and supply forecasting [146,147], reinforcement learning for logistics optimization [148,149,150], and embedded or edge AI systems deployed on microcontrollers for real-time contaminant or environmental monitoring [151]. More recent frameworks integrate AI with blockchain, IoT, RFID, IPFS, or Federated Learning (FL) to enable privacy-preserving and cross-organizational decision support [152,153,154,155]. Collectively, these studies confirm the growing maturity of AI-driven traceability solutions in agri-food systems; most remain at an experimental or pilot level. Recurrent challenges include small and fragmented datasets, limited scalability, and the absence of standardized evaluation frameworks, which restrict consistent benchmarking across AI approaches [156,157]. Despite promising results, a significant gap persists in the availability of large, labeled datasets specifically curated for counterfeit detection, limiting the development of robust and generalizable models [158]. Furthermore, integration challenges arise when combining AI with blockchain, IoT, and RFID technologies, often leading to interoperability issues and increased system complexity [130,159,160]. Explainability and transparency also remain major barriers to regulatory acceptance, as many AI systems function as black boxes with limited interpretability. Few studies have incorporated ethical and explainability principles directly into their design, leaving trust and compliance concerns largely unresolved. The use of AI in agri-food traceability raises additional ethical considerations related to data privacy, algorithmic transparency, and potential bias [161,162]. Large datasets collected through IoT devices and sensors often include sensitive operational or environmental information from farms and supply chain actors [138,163]. Without adequate safeguards, this data could be exposed to unauthorized entities, creating privacy and competitive risks [139]. Addressing these issues requires focused efforts on dataset curation, the development of explainable and ethically aligned AI techniques, and ensuring interoperability with existing infrastructures. Optimizing lightweight AI models for resource-constrained edge devices is equally essential for practical deployment. These improvements are crucial for translating current technical progress into sustainable, trustworthy, and industry-ready solutions. Overall, AI has the potential to strengthen traceability in AFSCs through real-time data analysis, predictive modeling, and decision support. When effectively integrated with IoT, blockchain, and other digital tools, it can significantly enhance transparency, anti-counterfeiting capability, and operational efficiency. However, the effectiveness of AI-driven TSs is tightly coupled to the quality and reliability of upstream identity and sensing layers, as well as to integrity-layer mechanisms that ensure data trustworthiness, without which AI models risk producing unreliable or non-compliant outcomes. The findings are summarized in Table 7.

AI technologies constitute the intelligence layer of the proposed TRAC framework by transforming raw traceability data into predictive insights, anomaly detection mechanisms, and automated decision-support functions. Unlike blockchain or identification technologies, AI does not directly guarantee data integrity or capture physical events; its reliability depends fundamentally on the quality and completeness of upstream sensing and identity layers. From an operational perspective, AI enhances forecasting, fraud detection, quality grading, and process optimization, thereby strengthening anti-counterfeiting and transparency objectives. However, practical deployment is constrained by data scarcity, model generalization limits, computational requirements, explainability concerns, and regulatory acceptance barriers. AI should be regarded as an enabling intelligence mechanism that complements sensing, identity, and integrity infrastructures within integrated, multi-layer traceability architectures.

## 10. Discussion

This section discusses the main findings of the review through a system-level interpretation of TRAC technologies in AFSCs. Rather than reiterating individual technology descriptions, the discussion synthesizes results across studies using the proposed five-layer TRAC framework to highlight functional roles, interdependencies, architectural patterns, and practical deployment implications. The discussion is organized into thematic subsections that examine layered system behavior, technology integration strategies, comparative trade-offs, and operational constraints relevant to real-world adoption.

### 10.1. Layered Interpretation of TRAC Architectures

**Figure 12:**
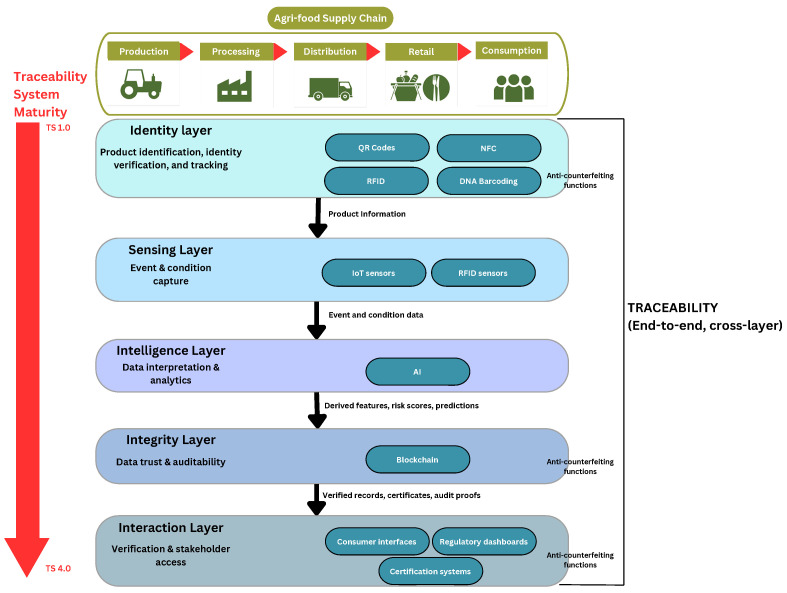
Layered architecture of end-to-end agri-food TRAC, mapping identity, sensing, intelligence, integrity, and interaction layers across the supply chain maturity spectrum (TS 1.0–TS 4.0).

Figure 12 synthesizes the reviewed literature by mapping TRAC technologies onto a unified five-layer TRAC framework composed of identity, sensing, intelligence, integrity, and interaction layers [138,165]. Rather than treating technologies as isolated solutions, this layered interpretation enables a system-level understanding of how traceability functionality emerges through coordinated interaction among heterogeneous components [166]. The identity layer provides the foundational linkage between physical products and digital records through technologies such as QR codes, RFID, NFC, and DNA barcoding [63,167]. These mechanisms enable product identification, origin attribution, and basic trace-back capabilities, forming the entry point for all downstream traceability processes. The sensing layer builds upon this foundation by capturing environmental, logistical, and process-related events through IoT sensors and monitoring devices [137,138]. As evidenced across the reviewed systems, sensing-layer data quality plays a critical role in overall system robustness, as incomplete or inaccurate measurements propagate upward and directly affect intelligence-layer analytics and integrity-layer guarantees [139,168]. The intelligence layer transforms raw sensing and identity-linked data into actionable insights using analytical models and AI-based techniques [165]. This layer supports predictive functions such as anomaly detection, quality assessment, and risk forecasting, thereby extending traceability beyond passive record-keeping toward decision support. The integrity layer addresses data trust, immutability, and auditability, most commonly through blockchain-based mechanisms [101]. By securing traceability records against manipulation and enabling verifiable audit trails, this layer underpins trust among supply chain stakeholders and regulatory authorities. Finally, the interaction layer provides access, verification, and governance mechanisms through dashboards, mobile applications, certification platforms, and consumer-facing interfaces, enabling independent validation and transparency [169]. Across the reviewed systems, traceability data typically originates from identity-linked sensing events, is processed through intelligence-layer analytics, secured via integrity mechanisms, and ultimately exposed to stakeholders through interaction-layer interfaces. This end-to-end data lifecycle highlights traceability as an inherently cross-layer capability that depends on the coordinated operation of all five layers [166]. Importantly, Figure 12 clarifies a conceptual distinction between TRAC within the proposed framework. While traceability requires end-to-end integration across identity, sensing, intelligence, integrity, and interaction layers, anti-counterfeiting operates as a more targeted, cross-cutting function primarily anchored in the identity, integrity, and interaction layers [63,101]. Robust identity verification, tamper-resistant record management, and independent stakeholder verification are sufficient to enable basic anti-counterfeiting, even in the absence of continuous sensing or advanced analytics. However, comprehensive traceability outcomes cannot be achieved unless all layers are coherently integrated. The reviewed evidence further confirms that no single technology independently delivers complete TRAC functionality [138]. Systems implementing only one or two layers typically exhibit fragmented visibility or weak trust guarantees. In contrast, multi-layer architectures that integrate identity, sensing, intelligence, and integrity components demonstrate higher robustness, improved fraud detection capability, and greater stakeholder confidence [165]. This layered complementarity confirms that TRAC should be interpreted as a system-of-systems problem rather than a single-technology solution. Within the proposed framework, TSs1.0–4.0 should be interpreted as progressive maturity stages rather than alternative architectures. TS 1.0 corresponds to identity-centric traceability, where product identification and basic record linkage dominate [166]. TS 2.0 emerges with the integration of sensing-layer technologies that enable event capture and condition monitoring across supply chain stages [138]. TS 3.0 reflects the incorporation of intelligence-layer capabilities, where analytics and AI transform raw traceability data into predictive and decision-support functions [165]. TS 4.0 represents fully mature TSs in which integrity and interaction layers ensure data immutability, auditability, and independent stakeholder verification [101]. In this sense, the five-layer TRAC framework provides the architectural structure, while TS 1.0–4.0 describe increasing levels of functional completeness achieved through cumulative layer integration, consistent with existing traceability maturity models in the literature.

### 10.2. System-Level Design Patterns and Layer Combinations

Beyond individual technologies, the reviewed studies summarized in Section 5, Section 6, Section 7, Section 8 and Section 9 reveal recurring system-level architectural design patterns that emerge from different combinations of the five TRAC layers [166,170]. These patterns reflect how TRAC objectives are operationalized under varying technical, economic, and organizational constraints. A first recurring pattern is the identity–interaction–integrity configuration [67,73], commonly adopted in consumer-facing TRAC applications. In this pattern, identity technologies such as QR codes, RFID, NFC, or DNA markers establish the link between physical products and digital records, integrity mechanisms (most frequently blockchain-based) ensure immutability and auditability of stored information, and interaction-layer interfaces enable verification by consumers, regulators, or certification bodies. This architecture is particularly effective for authenticity verification and fraud deterrence, as it supports independent validation without requiring continuous sensing or advanced analytics. However, its traceability depth remains limited, as it relies primarily on discrete, manually triggered events rather than continuous monitoring. A second prevalent pattern integrates identity–sensing–intelligence layers [146] to support operational traceability and quality monitoring across production, storage, and transportation stages. In these systems, IoT sensors continuously capture environmental and logistical data, while intelligence-layer components apply analytics or AI models to detect anomalies, predict quality degradation, or optimize handling conditions. This configuration enables rich, time-dependent visibility and proactive decision-making, particularly for perishable products. Nevertheless, without complementary integrity mechanisms, data trust remains vulnerable to manipulation, loss, or ambiguity, limiting its effectiveness for regulatory enforcement or dispute resolution. A third, more comprehensive pattern combines identity–sensing–intelligence–integrity layers [147,153] into end-to-end traceability architectures. These systems link continuous sensing data to product identities, apply analytics for interpretation, and secure resulting records through blockchain or cryptographic mechanisms. Such multi-layer integration supports both TRAC objectives by ensuring data continuity, analytical insight, and tamper resistance. While technically robust, this pattern introduces higher costs, infrastructure complexity, and governance requirements, which often constrain adoption by smallholders and small-scale enterprises. Across these patterns, the reviewed evidence indicates that traceability effectiveness increases with deeper layer integration, whereas anti-counterfeiting effectiveness depends primarily on the coordinated operation of identity, integrity, and interaction layers. Sensing and intelligence layers enhance traceability completeness and fraud detection sensitivity, but do not independently guarantee authenticity or trust. These observations demonstrate that TRAC performance is determined not by individual technologies, but by how functional layers are combined and aligned with specific supply chain objectives and deployment contexts. Layer importance also varies across supply chain stages, with identity mechanisms dominating early production and packaging, sensing layers playing a central role during transportation and storage, and interaction-layer interfaces becoming most critical at retail and consumer-facing points.

### 10.3. Error Propagation and Cross-Layer Dependencies

From a sensing-engineering perspective, the reliability of TRAC systems is fundamentally constrained by measurement-level uncertainties at the sensing layer. Across the reviewed studies, common issues include sensor measurement error, calibration drift over time, missing or irregularly sampled data, timestamp misalignment across distributed sensing nodes, and signal degradation due to environmental interference, power instability, or communication loss [137,168,171]. These factors directly affect data completeness, temporal consistency, and semantic accuracy before any higher-layer analytics or integrity mechanisms are applied [138,139]. An important system-level insight emerging from the layered analysis is the propagation of errors across TRAC layers, which can significantly affect overall system reliability. Errors introduced at lower layers, particularly within the sensing and identity layers, tend to cascade upward and amplify their impact at higher functional levels [168]. Inaccurate sensor measurements, calibration drift, missing data, or timestamp misalignment at the sensing layer directly compromise the quality of inputs used by intelligence-layer analytics, leading to erroneous predictions, false alarms, or undetected anomalies [172,173]. When such flawed outputs are subsequently committed to integrity-layer mechanisms, such as blockchain-based ledgers, incorrect information becomes permanently recorded, thereby reinforcing rather than correcting upstream errors [174]. Similarly, weaknesses in identity-layer mechanisms, including mislabeling, tag duplication, or physical substitution, can undermine the trustworthiness of otherwise robust sensing, intelligence, and integrity infrastructures. In these cases, the system may reliably process and secure data that are fundamentally associated with an incorrect product identity, resulting in traceability records that are technically consistent yet semantically invalid [138]. At the interaction layer, these compounded errors ultimately affect stakeholder decision-making, potentially misleading consumers, regulators, or certification bodies despite the presence of advanced verification interfaces. These observations highlight that TRAC system robustness cannot be achieved through strengthening individual layers in isolation. Instead, effective deployment requires coordinated error management strategies across layers, including sensor validation and redundancy, identity verification safeguards, confidence-aware analytics, and governance mechanisms that allow erroneous records to be flagged, contextualized, or corrected [168,171]. The layered framework thus enables not only functional classification, but also systematic reasoning about failure modes and reliability constraints in real-world TRAC systems. Table 8 summarizes the dominant error sources observed across TRAC layers and highlights how failures introduced at lower layers systematically propagate and amplify across the system.

### 10.4. Comparative System-Level Evaluation of TRAC Technologies

Beyond individual technology descriptions, effective TRAC technologies in AFSCs require a system-level evaluation [138,166] of how different technologies contribute to functional coverage, operational feasibility, and trustworthiness when deployed in real-world contexts. The layered TRAC framework enables such a comparison by shifting the focus from isolated technological capabilities to their functional roles within identity, sensing, intelligence, integrity, and interaction layers, as well as their combined contribution to end-to-end system performance. From this perspective, identity-layer technologies such as QR codes, RFID, NFC, and DNA barcoding primarily establish the linkage between physical products and digital records. QR codes and NFC provide low-cost, highly scalable mechanisms for consumer-facing verification and information access [175,176], but offer limited intrinsic protection against counterfeiting unless combined with secure backend systems. RFID enhances automation and real-time tracking capabilities across logistics and storage stages, improving traceability effectiveness, though its security depends on tag type and system integration. DNA barcoding delivers strong authenticity guarantees at the biological level, making it highly effective for anti-counterfeiting, but its reliance on specialized laboratory analysis limits scalability and reuse potential [177]. Sensing-layer technologies, dominated by IoT-based systems, enable continuous monitoring of environmental and operational conditions throughout the supply chain. These technologies substantially enhance traceability effectiveness by generating time-dependent data streams, particularly for perishable products. However, sensing infrastructures introduce additional costs [178,179] and reliability constraints, and their value depends critically on integration with higher-layer intelligence and integrity mechanisms. Intelligence-layer solutions, primarily based on AI, amplify the value of collected data by enabling automated interpretation, anomaly detection, and predictive decision support. While AI systems are highly reusable and scalable across applications [180,181,182,183], their effectiveness is fundamentally constrained by the quality and completeness of upstream identity and sensing data. Integrity-layer technologies, most notably blockchain-based systems, address trust, immutability, and auditability of traceability records. When integrated with robust identity and sensing mechanisms, blockchain significantly strengthens counterfeit protection and regulatory compliance. However, high deployment and operational costs [184,185,186], along with infrastructure complexity, limit feasibility for smallholders and resource-constrained environments. Importantly, integrity mechanisms alone cannot compensate for inaccurate or unreliable upstream data, reinforcing the system-of-systems nature of TRAC architectures. Table 9 synthesizes these trade-offs through a comparative system-level evaluation based on five criteria: cost, scalability, reuse potential, traceability effectiveness, and counterfeit protection. The qualitative ratings (very low to very high) were assigned based on a synthesis of evidence reported across the reviewed studies, reflecting typical deployment characteristics, functional layer coverage, and practical constraints observed in real-world implementations, rather than isolated experimental results or predefined quantitative thresholds. As such, the table should be interpreted as representing typical system-level behavior within AFSCs. The comparison highlights that no single technology independently satisfies all TRAC requirements. Low-cost identity technologies favor adoption and scalability but require integrity mechanisms to ensure trust. Sensing technologies provide rich operational visibility but introduce reliability and governance challenges without secure data management. Intelligence technologies enhance system adaptability and decision-making but depend critically on upstream data quality. Consequently, architectures that integrate complementary technologies across multiple layers generally demonstrate higher robustness and stakeholder confidence than isolated deployments. This analysis confirms that effective TRAC implementation is inherently a multi-layer, system-level challenge rather than a single-technology solution.

### 10.5. Practical Deployment Implications for SMEs and Smallholders

Small and Medium-sized Enterprises (SMEs) and smallholder farmers constitute the majority of actors within AFSCs; however, they operate under structural financial, technological, and organizational constraints that strongly condition the feasibility of TRAC adoption. Empirical analyses of SME growth and innovation capacity consistently show that limited access to capital, constrained investment capability, and uneven digital transformation significantly restrict the adoption of advanced technological infrastructures [187,188,189]. In agricultural contexts, innovation potential is often unevenly distributed, with many medium-sized and small-scale enterprises lacking the financial and organizational readiness required for high-complexity digital systems [189,190,191]. Consequently, TRAC architectures originally designed for vertically integrated or capital-intensive supply chains are frequently impractical in SME-dominated environments. From a system-level perspective, SME-oriented TRAC deployments therefore tend to favor lightweight and modular architectures that prioritize affordability, operational simplicity, and incremental scalability rather than fully automated end-to-end integration [187,192]. Within the proposed five-layer TRAC framework, this typically manifests as architectures centered primarily on the identity and interaction layers, complemented by selectively deployed sensing, intelligence, and integrity components according to available economic and technical capacity. Low-cost identity technologies such as QR codes, NFC tags, or basic RFID labels are particularly compatible with SME constraints, as they provide immediate product–data linkage with minimal infrastructure requirements and low operational overhead. Such incremental adoption aligns with broader findings in SME innovation studies, which emphasize phased technological integration and reliance on shared digital platforms rather than in-house system development [187,189]. Based on the reviewed studies and the structural characteristics of SMEs documented in the uploaded references, a representative SME-oriented reference architecture can be identified. This architecture consists of: (i) identity-layer mechanisms relying on QR codes or low-cost RFID for product identification and basic trace-back, (ii) selective sensing-layer integration, where environmental or logistical monitoring is applied at critical control points rather than continuously, (iii) cloud-based or externally hosted intelligence services, where analytics and decision-support functions are executed off-site to reduce local computational and maintenance burdens, and (iv) lightweight integrity mechanisms, such as permissioned blockchain services or secure centralized ledgers operated by cooperatives, certification bodies, or supply-chain aggregators rather than by individual producers. Interaction-layer interfaces, typically implemented through mobile applications or web dashboards, play a central role by enabling consumer verification and regulatory reporting without requiring advanced technical expertise from farmers. This distributed responsibility model is consistent with SME development research, which highlights the importance of ecosystem-level support, shared infrastructure, and intermediary institutions in enabling technological upgrading [188,189]. The primary advantages of this architecture lie in its reduced entry cost, modularity, and compatibility with existing SME workflows. Producers can initially adopt identity and interaction layers to meet regulatory or market-driven traceability requirements, while progressively integrating sensing, intelligence, or integrity components as financial capacity, digital literacy, and organizational coordination improve [187]. However, these architectures also present limitations, including reduced traceability depth, dependence on external service providers, and potential fragmentation when supply chain participation is incomplete. These constraints reflect broader structural barriers to SME digital transformation identified in the uploaded studies, including financing gaps, limited absorptive capacity, and uneven innovation diffusion across agricultural enterprises [188,189]. Overall, the evidence indicates that effective TRAC deployment in SME and smallholder environments is not primarily a matter of selecting individual technologies, but of designing system architectures that balance functional layer coverage with financial feasibility, innovation readiness, and institutional support capacity. The proposed five-layer TRAC framework therefore provides a structured lens for guiding incremental and economically realistic adoption pathways, enabling SMEs to progress toward more comprehensive TRAC capabilities as organizational maturity increases.

## 11. Future Research Directions

Based on the reviewed technologies and identified practical challenges, key research directions include:

### 11.1. Enhancing Integration of Technologies

Future research should prioritize the design of low-cost, interoperable, and modular traceability architectures that integrate IoT, AI, blockchain, RFID, and QR codes into cohesive TRAC systems rather than isolated technological solutions. A persistent challenge in agri-food environments, particularly in rural or remote regions, is the limited availability of reliable internet connectivity. One promising direction involves deploying AI models directly on IoT devices, enabling localized sensing, anomaly detection, and real-time quality classification at the edge. This approach transforms IoT nodes from passive data loggers into intelligent agents while reducing dependence on cloud infrastructure and continuous connectivity. Blockchain integration within such architectures should remain lightweight and purpose-driven. Instead of recording all traceability data on-chain, future systems should store only essential cryptographic proofs such as hashed identifiers, timestamps, or batch-level metadata to preserve data integrity and auditability while minimizing storage, energy, and latency overheads. Larger or sensitive datasets, including raw sensor streams or images, can remain off-chain, with blockchain functioning primarily as a verification index rather than a primary data repository. Low-cost identification technologies play a complementary role in integrated systems. QR codes offer an accessible and scalable gateway to traceability information, enabling stakeholders and consumers to verify product data using standard smartphones. RFID tags can be optimized by storing only minimal identifiers that link to external databases, avoiding unnecessary memory and cost increases while maintaining automated tracking capabilities. For high-value and frequently counterfeited products, DNA barcoding remains one of the most reliable methods for detecting substitution and origin fraud. However, its high cost, laboratory dependence, and slow processing limit large-scale deployment. Future research should therefore explore AI-based anti-counterfeiting approaches trained on multimodal datasets such as images, chemical sensor outputs, or packaging metadata as scalable, field-ready alternatives that may approximate DNA-level verification in selected use cases at significantly lower cost. Seamless integration across heterogeneous devices and platforms further requires the adoption of standardized communication protocols and lightweight middleware, including MQTT and REST APIs, to support interoperability across the agri-food ecosystem. For constrained or low-connectivity environments, protocols such as LoRaWAN, CoAP, and Bluetooth Low Energy offer energy-efficient and context-appropriate communication, ensuring continuity of traceability even under challenging operational conditions. Within the proposed five-layer TRAC framework, this research direction primarily strengthens the identity, sensing, and intelligence layers, while enabling effective coupling with integrity mechanisms to support end-to-end, system-level traceability.

### 11.2. Improving Scalability and Affordability

To enable widespread adoption of TRAC systems across AFSCs, particularly by small- and medium-scale producers. Future research must address scalability as both an economic and organizational challenge, rather than focusing solely on technological feasibility. A key direction involves the use of low-cost Integrated Circuits (ICs) and programmable edge devices for local data collection and preprocessing, which can significantly reduce hardware costs and energy consumption while supporting incremental system expansion. When combined with edge–cloud hybrid architectures, such designs ensure that only essential, distilled information is transmitted to the cloud, lowering bandwidth demands and improving operability in regions with limited or unreliable connectivity. Affordability can be further improved through the selective use of minimalist identification technologies. QR codes, for example, offer a highly cost-effective mechanism for linking physical products to digital traceability records accessible via smartphones, enabling broad participation without specialized equipment. Where automated tracking is required, passive RFID tags configured to store only critical identifiers can provide scalable identification while limiting per-unit costs. The widespread availability of mobile phones as universal access devices further reduces adoption barriers by allowing producers, intermediaries, and consumers to interact with TSs using familiar tools. However, scalability-driven cost reduction introduces important trade-offs that must be explicitly managed. Low-cost sensors may exhibit reduced accuracy, robustness, or lifespan, potentially compromising data quality and undermining trust. Similarly, modular or vendor-diverse systems can create interoperability challenges that hinder long-term scalability. Cooperative infrastructures such as shared gateways, data hubs, or community managed platforms offer promising cost-sharing models but require clear governance mechanisms to ensure fairness, data security, and sustainable operation. Many of the reviewed RFID-, blockchain-, and QR-based solutions remain at prototype or pilot stages, underscoring the need for longitudinal scalability studies conducted in real production environments. Future research should therefore focus on balancing affordability with reliability through mechanisms such as error-tolerant data processing for low-grade sensors, standardized modular interfaces to ensure compatibility, and cooperative business models that align economic incentives across stakeholders. Within the proposed TRAC framework, this direction primarily reinforces the identity and interaction layers, while ensuring that cost-effective system scaling does not compromise data integrity or stakeholder trust.

### 11.3. Addressing Ethical and Privacy Concerns

As TSs increasingly rely on digital technologies such as blockchain, AI, IoT, and RFID, concerns related to data ownership, consent, privacy, and accountability have become central to their long-term legitimacy and adoption. Small- and medium-scale farmers, often the primary generators of operational data, may have limited visibility into how information related to planting schedules, environmental conditions, or production practices is collected, shared, and ultimately exploited. Without clear governance mechanisms, such asymmetries risk undermining trust and discouraging participation in traceability initiatives. Future research should therefore prioritize the development of transparent data governance frameworks that explicitly define data ownership, access rights, usage conditions, and consent management across the AFSC. A promising direction involves the adoption of Data Space architectures, where data exchange occurs under clearly defined contractual rules, technical standards, and usage policies. Within such frameworks, farmers can retain control over their data while selectively sharing, selling, or licensing anonymized datasets to third parties such as processors, certification bodies, regulators, or AI research institutions, thereby fostering a data economy in which producers act as active stakeholders rather than passive data sources. From a technical perspective, privacy protection must be embedded into system design. In blockchain- and cloud-based TSs, off-chain storage of sensitive information, linked through cryptographic hashes, can reduce public exposure while preserving verifiability and auditability. In parallel, future work should explore privacy-preserving AI techniques, including FL, homomorphic encryption, and differential privacy, to enable the training of ML models on distributed, farm-level data without centralizing raw information. Such approaches support cross-regional pattern discovery and predictive analytics while respecting individual privacy and regulatory constraints. Ultimately, balancing transparency with confidentiality requires a combination of robust technical safeguards, enforceable legal frameworks, and user-oriented tools that allow stakeholders, particularly small-scale producers, to make informed decisions about how their data is accessed and monetized. Within the proposed five-layer TRAC framework, this research direction primarily strengthens the integrity and interaction layers, ensuring that TSs not only function technically, but also establish durable trust, accountability, and social acceptance.

### 11.4. Advancing Deployment Readiness

While many technologies proposed for agri-food traceability demonstrate strong potential, a persistent gap remains between controlled pilot studies and sustained real-world deployment. In this review, TRLs proved to be a valuable tool for assessing not only the functional maturity of individual solutions, but also their proximity to operational use within real agri-food environments. TRL assessment helps distinguish between systems that are technically feasible in laboratory or simulated settings and those that can withstand the environmental, infrastructural, and organizational constraints encountered in practice. Several reviewed approaches, particularly blockchain and DNA-based systems, are frequently validated through simulations or laboratory-scale pilots, leaving their real-world performance largely untested. Future research should therefore adopt TRL-based evaluation as a standard assessment practice, while extending it beyond technical functionality to include contextual indicators such as usability under low-connectivity conditions, ease of maintenance, energy requirements, and training needs for non-expert users. These dimensions are critical for evaluating whether a solution can realistically be deployed and maintained by diverse actors across AFSCs. Achieving higher TRLs requires systematic testing in operational agri-food settings, rather than exclusively in academic or experimental environments. Collaborative field trials involving farms, cooperatives, logistics operators, and supply chain hubs should be prioritized to assess system performance under realistic conditions, including weather variability, intermittent power supply, heterogeneous infrastructure, and varying levels of digital literacy. Such evaluations provide essential feedback for refining system robustness, usability, and long-term sustainability. In parallel, future work should aim to define deployment standards and reference architectures that guide practitioners in adapting traceability solutions to different crops, supply chain scales, and regulatory contexts. These may include best practices for sensor placement, data acquisition frequency, cloud–edge integration strategies, and fallback mechanisms to preserve data continuity during network outages. Early involvement of end-users in testing and validation processes further ensures alignment with existing workflows and increases the likelihood of sustained adoption beyond the project lifecycle. Within the proposed five-layer TRAC framework, TRL-driven validation supports coordinated maturation across the sensing, intelligence, integrity, and interaction layers, positioning TRL not merely as a classification tool, but as a strategic framework for responsible and deployable traceability innovation.

### 11.5. Data Foundations and Climate-Resilient Traceability Systems

The effectiveness of AI-driven TRAC systems in AFSCs critically depends on the availability of large-scale, high-quality, and context-diverse datasets that reflect real operational conditions. Current datasets remain limited in scope, often underrepresenting specific commodities, climatic regions, and fraud scenarios. Future research should therefore prioritize the development of structured, multimodal datasets tailored to TRAC, incorporating data such as packaging and product images, QR and RFID identifiers, chemical and environmental sensor outputs, and metadata describing origin, authentication status, and tampering indicators. Unlike generic traceability records, these datasets must capture subtle variations between genuine and counterfeit products under diverse environmental and logistical conditions to support the development of models that generalize beyond controlled laboratory settings. Dataset development, however, faces significant practical barriers. Collecting representative samples across regions and seasons is costly and time-intensive, while producers may be reluctant to share data due to privacy concerns or competitive risks. Although crowdsourced annotation can accelerate labeling, it introduces risks of bias and inconsistency, and benchmark datasets require sustained institutional commitment to remain relevant. Future work should therefore explore incentive-based data sharing models, such as data cooperatives that reward contributors, alongside anonymization and governance frameworks that enable data reuse without compromising confidentiality. International collaborations and open-access repositories can further support benchmarking, transparency, and reproducibility, accelerating progress across heterogeneous agri-food systems. In parallel, traceability infrastructures must be designed to remain reliable under climate variability and operational disruption, including extreme weather events, transport delays, power instability, and intermittent connectivity. Rather than relying on uniform technological deployments, future systems should be context-aware and regionally adapted, accounting for local infrastructure, environmental risks, and energy availability. In low-connectivity or disaster-prone environments, local data storage with deferred synchronization allows traceability processes to continue uninterrupted, while energy-efficient devices potentially supported by solar power or battery backups enhance system autonomy. Integrated environmental sensing can further enable sensor-driven quality checkpoints that detect spoilage risks during storage and transport under adverse conditions. A key research priority is ensuring data continuity across all supply chain nodes, even during disruptions. This can be achieved through redundant mechanisms such as offline-capable QR or NFC verification, minimal RFID tracking, and localized data gateways that preserve traceability when networks fail. Systems must also support post-disruption data reconciliation to restore transparency and auditability. Within the proposed five-layer TRAC framework, robust data foundations and climate-resilient design primarily reinforce the sensing and intelligence layers, while supporting integrity and interaction through reliable data continuity. Together, these directions enable TSs that are not only accurate and intelligent, but also resilient to the environmental and operational realities increasingly shaping global AFSCs.

### 11.6. Transforming Consumer Roles in Traceability

Consumers should no longer be viewed merely as passive recipients of traceability information, but rather as active participants and verification nodes within TRAC systems. Through interactions with traceability platforms such as scanning QR codes or tapping NFC tags, consumers initiate data flows that confirm product presence, validate authenticity, and generate real-world evidence of product movement. This transforms the consumer from an endpoint into a human-in-the-loop verification mechanism, enabling TSs to close the informational loop between production, distribution, and final consumption. Crucially, consumer interactions can be propagated upstream to reinforce system integrity. When a product scan confirms authenticity at the point of purchase, producers and supply chain actors can be notified that the item has reached its intended destination without substitution. Conversely, anomalous interaction patterns such as duplicate identifiers, inconsistent geographic locations, or repeated consumer reports of quality issues can act as early indicators of counterfeiting, diversion, or unauthorized redistribution. In this sense, consumer participation contributes not only to transparency, but also to distributed anomaly detection across the supply chain. To encourage engagement, future systems may incorporate incentive mechanisms such as loyalty rewards, educational content, or verifiable acknowledgments linked to blockchain-based records. However, consumer-driven traceability also introduces non-trivial challenges. Participation levels depend on awareness, motivation, and access to digital tools, which vary across regions and demographics. Moreover, automated or fraudulent scans can introduce noise into traceability records, undermining data reliability if left unchecked. Future research should therefore develop scan-verification and validation protocols capable of distinguishing genuine human interactions from automated or malicious activity. Equally important are the privacy implications of consumer participation. Scan histories may reveal sensitive consumption patterns or behavioral profiles if improperly managed. As such, consumer-generated data should be subject to strict minimization, anonymization, and consent-aware handling. Within the proposed five-layer TRAC framework, consumers operate primarily within the interaction layer, while their verified actions reinforce the integrity layer by providing decentralized, real-world confirmation of traceability claims. Designing consumer participation as a core system function—rather than an optional interface represents a critical research direction for building trustworthy, scalable, and socially grounded traceability infrastructures.

### 11.7. Using Advanced Analytics to Reduce Food Waste

Although reducing food waste is not a primary focus of this review, it is consistently discussed in the agri-food and the food safety literature as a relevant secondary outcome of effective traceability and data-driven supply chain management. Food waste occurs at multiple stages of the AFSC, from harvesting and storage to transportation, retail, and final consumption, and is often exacerbated by limited visibility into product condition, timing, and handling history. In this context, TSs enriched with advanced analytics and AI can indirectly contribute to waste reduction without shifting the analytical focus away from TRAC. By leveraging continuous and verified traceability data, such as temperature and humidity profiles, location trajectories, transport duration, and batch history, AI models can generate more accurate shelf-life predictions and dynamic freshness indicators. These insights support improved decision-making across the supply chain, including optimal harvest timing, logistics planning, and inventory rotation. For example, farmers can identify appropriate harvest windows by jointly considering crop maturity, forecasted weather conditions, downstream transport availability, and expected market demand. Similarly, analytics-driven routing and cold-chain management can prioritize faster delivery or enhanced preservation for high-risk batches. At the distribution and retail levels, traceability-informed analytics enable first-expiring-first-out (FEFO) strategies, adaptive pricing, and targeted redistribution practices aimed at preventing unnecessary disposal. Moreover, when TSs detect exposure to suboptimal storage or transport conditions, automated alerts can trigger early mitigation actions, such as rerouting, accelerated sales, or quality inspections, before spoilage occurs. Importantly, the effectiveness of these predictive approaches depends on the integrity and continuity of traceability data, as incomplete or unreliable records undermine analytical accuracy. Future research should therefore focus on integrating advanced predictive analytics tightly with real-time traceability infrastructures, ensuring that decision-support models operate on trusted, end-to-end data rather than isolated observations. Within the proposed five-layer TRAC framework, this direction primarily strengthens the intelligence layer, while relying on robust sensing and integrity layers to enable actionable, sustainability-oriented outcomes across AFSCs.

## 12. Conclusions

This review examined the evolution and current landscape of TRAC technologies in AFSCs, with an emphasis on secure, scalable, and cost-effective system design. Rather than focusing on individual technologies in isolation, the review adopted a system-level perspective, analyzing how diverse technological solutions collectively support TRAC functions across different stages of AFSCs. By mapping these technologies within a unified conceptual framework, the review highlighted how TRAC capabilities emerge through the integration of identity, sensing, intelligence, integrity, and interaction mechanisms. A central contribution of this work lies in evaluating TRAC technologies not only in terms of technical functionality, but also with respect to their deployment readiness using the TRL framework. This analysis revealed that, despite significant technical progress, many proposed solutions remain constrained by practical limitations related to cost, interoperability, energy consumption, and infrastructure availability, particularly in rural and resource-constrained environments. These findings underscore the importance of assessing traceability solutions within realistic operational contexts rather than solely through laboratory or pilot-scale validations. The review further emphasized the growing role of decentralized intelligence, data integrity, and stakeholder participation in strengthening trust and transparency across AFSCs. Edge AI and cloud–edge hybrid architectures were identified as promising approaches to distribute analytical capabilities closer to data sources and reduce dependence on continuous connectivity, while blockchain and cryptographic mechanisms offer verifiable and tamper-evident records without requiring centralized control. In parallel, consumer participation and human-in-the-loop verification mechanisms were highlighted as increasingly important components of trustworthy TSs. A key practical insight emerging from the layered analysis is that the primary maturity gap in agri-food traceability does not stem from individual technologies themselves, but from the lack of coordinated, interoperable adoption across all five TRAC layers. In particular, smallholders often deploy identity or sensing solutions in isolation without complementary integrity and intelligence mechanisms, resulting in fragmented visibility and limited trust guarantees. Therefore, future deployments should prioritize integrated, multi-layer architectures rather than incremental single-technology adoption. Overall, the findings indicate that future TRAC systems must prioritize integrative, adaptive, and ethically governed architectures that align with the heterogeneous needs of agri-food stakeholders, from smallholder producers to regulators and end consumers. Achieving this goal requires a balanced synthesis of technological innovation, data governance, stakeholder inclusion, and real-world validation. By consolidating existing knowledge within a coherent framework and identifying persistent deployment challenges, this review provides a structured reference to support the design, evaluation, and implementation of next-generation TRAC systems suited to the complexity of global AFSCs.

## Figures and Tables

**Table 1:** Comparison between this review and recent survey articles published in 2025 on agri-food TRAC and related digital technologies.

Review (Year)	Main Scope	Primary Focus	Technologies Covered	Conceptual / System Framework	Cross-Technology Integration	Deployment Readiness / TRL	Consumer / Governance Aspects
This review (2025)	End-to-end AFSCs	TRAC	QR codes, RFID/NFC, IoT, AI/ML, blockchain, DNA-based methods	Explicit layered TRAC framework (identity, sensing, intelligence, integrity, interaction)	Strong: technologies analyzed as interdependent system layers	Explicit TRL-based assessment across all technologies	Explicit: consumer participation, data ownership, privacy, trust
Rossi et al. (2025) [14]	AFSCs	Traceability systems and innovation	IoT, AI, blockchain, sensing technologies	Traceability-oriented taxonomy	Moderate	Not explicitly addressed	Partial
Halder et al. (2025) [15]	AFSCs	Secure AI-enabled Industrial IoT	AI, IoT, security mechanisms	Taxonomy-based security framework	Limited	Partially discussed	Partial (security-focused)
Zhang et al. (2025) [16]	AFSCs	Farmer participation in traceability	Digital platforms, ICT systems	Socio-technical adoption framework	Limited	Not addressed	Explicit (farmer participation)
Plakantara et al. (2025) [17]	AFSCs	Digital transformation and food safety	IoT, AI, blockchain, big data	Transformation-oriented framework	Moderate	Not addressed	Partial
Verna et al. (2025) [18]	Food processing chains	Quality-driven traceability	Blockchain, IoT, RFID	Quality 4.0 perspective	Limited	Not addressed	Partial
Vasileiou et al. (2025) [19]	AFSCs	Blockchain-based traceability	Blockchain, smart contracts	Systematic review structure	Limited	Not explicitly addressed	Partial
Morchid et al. (2025) [20]	Agricultural systems	Smart agriculture and sustainability	IoT, blockchain, AI	Descriptive survey	Limited	Not addressed	Limited
Xue et al. (2025) [21]	AFSCs	Supply chain risk and resilience	Digital technologies (general)	Risk-oriented analytical framework	Limited	Not addressed	Limited

**Table 2:** Rule-based criteria used for TRL assignment in this review.

TRL	Assignment Criteria Applied in This Review
TRL 1	Basic principles discussed, no system architecture or implementation.
TRL 2	Conceptual framework or architecture proposed, no implemented components.
TRL 3	Algorithmic logic or proof-of-concept evaluated using simulations or synthetic data.
TRL 4	Prototype component or subsystem validated under laboratory conditions.
TRL 5	Integrated prototype validated in a relevant but controlled environment.
TRL 6	End-to-end system prototype demonstrated using real hardware and realistic workflows, without sustained real-world operation.
TRL 7	System prototype demonstrated in an operational agri-food environment with real products or stakeholders, limited in scope or duration.
TRL 8	System evaluated through extended operation, multiple production cycles, or longitudinal studies.
TRL 9	System proven in routine operation or production-grade deployment across organizations or sites.

**Table 3:** Summary of reviewed studies in Section 5.

Barcode, Non-Electronic Approaches and Molecular-Based TRAC			
Ref	Technology	Other Technologies	TRL
[67]	QR Code	Blockchain, cloud	5
[69]	QR Code	Mobile app, Web services	7
[70]	2D Barcodes	2D software reader, web services	4
[71]	QR Code	–	7
[72]	QR Code	Mobile app, Web app	6
[73]	QR Code	Mobile app, Web app, cloud	7
[74]	QR Code	Mobile app, Web app	5
[75]	DNA Barcoding	–	4
[76]	DNA Barcoding	–	4
[77]	DNA Barcoding	–	4
[78]	DNA Barcoding	–	5

**Table 4:** Summary of reviewed studies in Section 6.

RFID and NFC Approaches for Agri-Food TRAC			
Ref	Technology	Other Technologies	TRL
[85]	RFID	-	6
[86]	RFID	Blockchain	5
[87]	RFID	-	6
[88]	RFID	-	6
[89]	RFID	-	5
[90]	RFID	-	4
[91]	RFID	GPS sensors	4
[92]	NFC	Mobile app	6
[5]	RFID	GPS, mobile app, QR code	5
[94]	RFID	RSA public key cryptography	5
[93]	NFC	Mobile app,	7
[6]	RFID	Blockchain, QR code	5
[95]	RFID	-	6

**Table 5:** Summary of reviewed studies in Section 7.

Blockchain and ledger approaches for Agri-Food TRAC			
Ref	Technology	Other Technologies	TRL
[105]	Blockchain	IoT	5
[106]	Blockchain	IoT, Web apps	6
[107]	Blockchain	IoT, AI	5
[108]	Blockchain	IoT, IPFS	6
[109]	Blockchain	IoT, IPFS	5
[111]	Blockchain	RFID, sensors	8
[112]	Blockchain	QR code	6
[113]	Blockchain	IPFS, QR code	6
[114]	Blockchain	-	5
[115]	Blockchain	IoT, QR Code	6
[116]	Blockchain	QR code	5
[117]	Blockchain	RFID, sensors	6
[118]	Blockchain	QR Code	5
[119]	Blockchain	RFID, IoT, QR Code	4
[120]	Blockchain	-	7

**Table 6:** Summary of reviewed studies in Section 8.

IoT Sensors Approaches for Agri-Food TRAC			
Ref	Technology	Other Technologies	TRL
[129]	IoT	Web app	4
[130]	IoT	Blockchain, QR Code	5
[131]	IoT	Blockchain, QR Code	5
[132]	IoT	Blockchain	4
[133]	IoT	Blockchain	5
[134]	IoT	QR Code	5
[135]	IoT	Web and mobile apps	5
[136]	IoT	RFID	5

**Table 7:** Summary of reviewed studies in Section 9.

AI Approaches for Agri-Food TRAC			
Ref	Technology	Other Technologies	TRL
[144]	AI (CNN)	IoT	5
[145]	AI (CNN)	IoT	3
[146]	AI (XGBoost)	IoT, RFID	7
[147]	AI (RNN)	IoT, Blockchain	5
[148]	AI (DRL)	Blockchain	4
[149]	AI (DRL)	Blockchain and IoT	5
[150]	AI (DRL)	Blockchain, Mobile app	4
[151]	AI (NN)	IoT	5
[152]	AI	IoT, Blockchain	6
[153]	AI	IoT, Blockchain	4
[154]	AI (LLMs)	Blockchain	3
[155]	AI (TCNs, DL) and FL	Blockchain, QR codes	5

**Table 8:** Cross-layer error sources and propagation risks in TRAC systems.

TRAC Layer	Typical Error Sources	Immediate Impact	Propagation Risk to Higher Layers
Identity	Mislabeling, tag duplication, physical substitution, damaged or unreadable QR/RFID/NFC tags, DNA sampling errors	Incorrect linkage between physical product and digital record	Compromises sensing attribution, corrupts AI inference validity, and leads to immutable storage of incorrect identities in integrity layers
Sensing	Measurement noise, calibration drift, sensor aging, missing data, packet loss, timestamp misalignment, environmental interference	Incomplete, inaccurate, or temporally inconsistent data streams	Leads to false predictions, missed anomalies, unreliable AI outputs, and permanent storage of flawed data in blockchain-based ledgers
Intelligence	Model bias, overfitting, poor generalization, low-quality training data, lack of explainability	Incorrect anomaly detection, misclassification, unreliable forecasts	Erroneous decisions recorded as trusted outcomes, misleading stakeholders, and reduced regulatory confidence
Integrity	Irreversible recording of incorrect data, delayed synchronization, governance misconfiguration	Immutability of flawed or misleading records	Amplifies upstream errors by preventing correction, reinforcing false trust and complicating dispute resolution
Interaction	Poor interface design, misleading visualizations, lack of context, delayed updates	Incorrect interpretation by consumers, auditors, or regulators	Erodes trust, leads to incorrect decisions despite technically robust backend systems

**Table 9:** Comparison of TRAC technologies in agri-food systems based on cost, scalability, reuse potential, traceability effectiveness, and counterfeit protection.

Technology	Cost Rate	Scalability	Reuse Potential	Traceability Effectiveness	Counterfeit Protection
QR Codes	Very Low	High	High	Medium–High	Low
RFID	Medium	High	High	High	Medium
NFC	Medium	Low	High	Medium	Medium
IoT	Low–High	High	Medium	High	Medium
AI	High–Very High	High	High	High	High
DNA Barcoding	Very High	Low	Low	Very High	Very High
Blockchain	Very High	Medium	High	Very High	Very High

## Data Availability

No new data were created or analyzed in this study.

## Abbreviations

The following abbreviations are used in this manuscript:

TRAC	Traceability and Anti-counterfeiting
OFS-OSB	One-step-forward and one-step-backward
AFSC	Agri-Food Supply Chain
RFID	Radio-Frequency Identification
NFC	Near Field Communication
IoT	Internet of Things
AI	Artificial Intelligence
TRL	Technology Readiness Level
TS	Traceability System
EU	European Union
US	United States
FSL	Food Safety Law
CFDA	Chinese Food and Drug Administration
NHFPC	National Health and Family Planning Commission
RASFF	Rapid Alert System for Food and Fee
COOL	Country-of-Origin Labeling
IPFS	Interplanetary File System
DL	Deep Learning
FL	Federated Learning
NN	Neural Network
CNN	Convolutional Naural Network
RNN	Recurrent Neural Networks
DRL	Deep Reinforcement Learning
LLMs	Large Language Models
TCNs	Temporal Convolutional Networks
SME	Small and Medium-sized Enterprise
ICs	Integrated Circuits
M4C2	Mission 4 Component 2
